# Thirteen complete chloroplast genomes of the costaceae family: insights into genome structure, selective pressure and phylogenetic relationships

**DOI:** 10.1186/s12864-024-09996-4

**Published:** 2024-01-17

**Authors:** Dong-Mei Li, Yan-Gu Pan, Hai-Lin Liu, Bo Yu, Dan Huang, Gen-Fa Zhu

**Affiliations:** Guangdong Key Lab of Ornamental Plant Germplasm Innovation and Utilization, Environmental Horticulture Research Institute, Guangdong Academy of Agricultural Sciences, Guangzhou, 510640 China

**Keywords:** Costaceae, Chloroplast genome, Comparative genomics, Genome evolution, Phylogenetic relationships, Divergence time

## Abstract

**Background:**

Costaceae, commonly known as the spiral ginger family, consists of approximately 120 species distributed in the tropical regions of South America, Africa, and Southeast Asia, of which some species have important ornamental, medicinal and ecological values. Previous studies on the phylogenetic and taxonomic of Costaceae by using nuclear internal transcribed spacer (*ITS*) and chloroplast genome fragments data had low resolutions. Additionally, the structures, variations and molecular evolution of complete chloroplast genomes in Costaceae still remain unclear. Herein, a total of 13 complete chloroplast genomes of Costaceae including 8 newly sequenced and 5 from the NCBI GenBank database, representing all three distribution regions of this family, were comprehensively analyzed for comparative genomics and phylogenetic relationships.

**Result:**

The 13 complete chloroplast genomes of Costaceae possessed typical quadripartite structures with lengths from 166,360 to 168,966 bp, comprising a large single copy (LSC, 90,802 − 92,189 bp), a small single copy (SSC, 18,363 − 20,124 bp) and a pair of inverted repeats (IRs, 27,982 − 29,203 bp). These genomes coded 111 − 113 different genes, including 79 protein-coding genes, 4 rRNA genes and 28 − 30 tRNAs genes. The gene orders, gene contents, amino acid frequencies and codon usage within Costaceae were highly conservative, but several variations in intron loss, long repeats, simple sequence repeats (SSRs) and gene expansion on the IR/SC boundaries were also found among these 13 genomes. Comparative genomics within Costaceae identified five highly divergent regions including *ndhF, ycf1-D2*, *ccsA-ndhD*, *rps15-ycf1-D2* and *rpl16-exon2-rpl16-exon1*. Five combined DNA regions (*ycf1-D2* + *ndhF*, *ccsA-ndhD* + *rps15-ycf1-D2*, *rps15-ycf1-D2* + *rpl16-exon2-rpl16-exon1*, *ccsA-ndhD* + *rpl16-exon2-rpl16-exon1*, and *ccsA-ndhD* + *rps15-ycf1-D2* + *rpl16-exon2-rpl16-exon1*) could be used as potential markers for future phylogenetic analyses and species identification in Costaceae. Positive selection was found in eight protein-coding genes, including *cemA, clpP*, *ndhA*, *ndhF*, *petB*, *psbD*, *rps12* and *ycf1*. Maximum likelihood and Bayesian phylogenetic trees using chloroplast genome sequences consistently revealed identical tree topologies with high supports between species of Costaceae. Three clades were divided within Costaceae, including the Asian clade, *Costus* clade and South American clade. *Tapeinochilos* was a sister of *Hellenia*, and *Parahellenia* was a sister to the cluster of *Tapeinochilos + Hellenia* with strong support in the Asian clade. The results of molecular dating showed that the crown age of Costaceae was about 30.5 Mya (95% HPD: 14.9 − 49.3 Mya), and then started to diverge into the *Costus* clade and Asian clade around 23.8 Mya (95% HPD: 10.1 − 41.5 Mya). The Asian clade diverged into *Hellenia* and *Parahellenia* at approximately 10.7 Mya (95% HPD: 3.5 − 25.1 Mya).

**Conclusion:**

The complete chloroplast genomes can resolve the phylogenetic relationships of Costaceae and provide new insights into genome structures, variations and evolution. The identified DNA divergent regions would be useful for species identification and phylogenetic inference in Costaceae.

**Supplementary Information:**

The online version contains supplementary material available at 10.1186/s12864-024-09996-4.

## Background

Costaceae Nakai, commonly known as the spiral ginger family, comprises more than 120 species that are primarily native to the tropical regions of South America, Africa, and Southeast Asia [1–6]. It is one of the most easily recognizable family within the order Zingiberales by its well-developed and sometimes branched aerial shoots that have a characteristic spiral phyllotaxy and petaloid labellum formed by fusion of five sterile staminodes [1–6]. Some species of Costaceae can be used as garden ornamental plants and cut flowers [4, 7–8], some of them, such as *Costus speciosus*, can be used as medicinal plants for the treatment of inflammation, rheumatism, bronchitis, fever, headache, asthma, flatulence, constipation, helminthiasis, leprosy, skin diseases, hiccough, anemia, anticancer, as well as burning sensation on urination [4, 8–12], and some Neotropical *Costus* species can be used an ecological model to understand the mechanisms of biogeographic origins, floral evolution and Neotropical diversity [13–15].

Previous phylogenetic analyses of family Costaceae based on molecular (*ITS*, *trnL*-*F* and *trnK* including the *matK* coding region) and morphological data revealed three major clades with discrete biogeographic distribution: a South American clade, an Asian clade and an African-neotropical *Costus* clade (*Costus* clade) [2, 5, 16]. The South American clade comprised three genera *Chamaecostus*, *Dimerocostus* and *Monocostus*, the Asian clade included three genera *Cheilocostus*, *Paracostus* and *Tapeinochilos*, and the *Costus* clade consisted of New World *Costus*, African melittophilous *Costus*, and African *Costus* grade [2, 5, 16]. *Cheilocostus* was established to classify the Southeast Asian and Malesian species of the broadly defined genus *Costus* [5]. Four species, *C. speciosus* (≡ *Cheilocostus speciosus*), *C. lacerus* (≡ *Cheilocostus lacerus*), *C. globosus* (≡ *Cheilocostus globosus*), and *C. sopuenisis* (≡ *Cheilocostus sopuenisis*) were transferred to *Cheilocostus* in that study [5]. Although the genus *Cheilocostus* was native to South East Asia [5], its name and genetic status were disputed [5, 17–19]. Govaers [17] had proposed that *Cheilocostus* was an illegitimate superfluous name for *Hellenia*, and *Hellenia* should be taken up for this genus. This arrangement was met with some approvals [18, 19]. Recently, a phylogenetic tree based on an enlarged taxon sampling of the Asian clade has confirmed the paraphyly of *Hellenia* by using two chloroplast markers data (*trnK* intron and *trnL-F* spacer) [20]. Morphological analyses have suggested that members of the *Parahellenia* subclade differ from the *Hellenia* species in many characteristics [20]. Based on molecular and morphological evidence, the *Parahellenia* subclade has been recognized as a new genus [20]. However, all these phylogenetic trees of Costaceae encompassed multiple poor-resolution branches [2, 5, 16, 20]. In addition, the molecular evolution of complete chloroplast genomes from Costaceae containing species from the South American clade, Asian clade and *Costus* clade, remains poorly understood [2, 5, 13–15, 20]. Therefore, it is worthwhile to investigate phylogenetic relationships and molecular evolution of Costaceae which covers its three distributions sampling.

Chloroplasts are critical and dynamic organelles in plant cells for converting solar energy to carbohydrates through the process of photosynthesis and oxygen release [21, 22]. Chloroplast has its independent genome (chloroplast genome) in plant cells with a circular double-stranded DNA molecule, typically comprising a large single copy region (LSC), a small single copy region (SSC), and two copies of inverted repeats (IRa and IRb) [21, 22]. Compared with nuclear genomes, the chloroplast genomes are smaller in length, with less recombination and lower rates of nucleotide substitutions. Hence they have been widely utilized for studies on reconstructing phylogenetic relationships and molecular evolution from algae to higher plants [23–36]. With the rapid development of high-throughput sequencing technologies, it is now more accurate and more convenient to obtain complete chloroplast genomes. In recent years, although some chloroplast genome sequences of Costaceae have been reported [15, 20, 37], these genome sequences of most species studied were incomplete without comprehensive chloroplast genome analyses for Costaceae. Currently, the complete chloroplast genomes of the genus *Monocostus* in the South American clade are rare and much less than the Asian clade and *Costus* clade.

In this study, we newly sequenced, assembled and annotated complete chloroplast genomes of eight species of Costaceae (*Costus barbatus*, *C. beckii*, *C. dubius*, *C. woodsonii*, *C. speciosus* Guangdong, *C. speciosus* var. *marginatus*, *C. tonkinensis* Yunnan and *Monocostus uniflorus*) coming from the *Costus* clade, Asian clade and South American clade, respectively, and then performed comparative genomics and phylogenomics analyses by integrating five published complete chloroplast genomes of Costaceae from National Center for Biotechnology Information (NCBI). Our main aims were: (1) to characterize and to investigate these complete chloroplast genome structures and variations in Costaceae; (2) to detect variations of long repeats, simple sequence repeats (SSRs), and codon usage patterns of these chloroplast genomes in Costaceae; (3) to identify highly variable regions for potential DNA markers developing and to understand molecular evolution of chloroplast genomes in Costaceae; and (4) to reconstruct phylogeny and to assess the divergence time of Costaceae, especially, *Hellenia* and *Parahellenia* in the Asian clade.

## Results

### General characteristics of thirteen chloroplast genomes

**Fig. 1:**
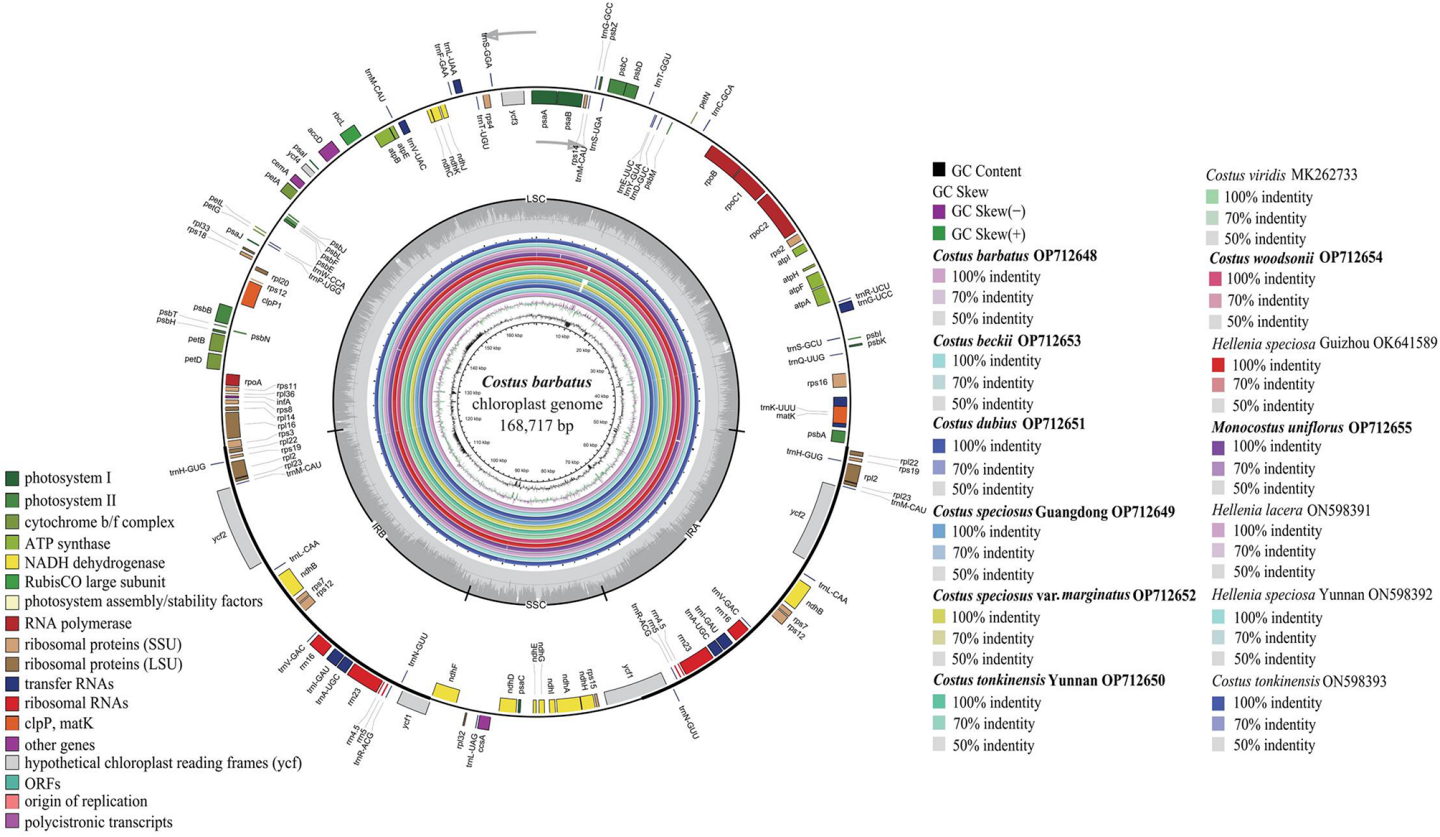
Chloroplast genome map of *C. barbatus* (GenBank accession number: OP712648; the outermost three rings) and CGView comparison of thirteen complete chloroplast genomes in the Costaceae family (the inter rings with different colors). Genes shown on the outside of the outermost first ring are transcribed counter-clockwise and on the inside clockwise. Outermost second ring with darker gray corresponds to GC content, whereas outermost third ring with the lighter gray corresponds to AT content of *C. barbatus* chloroplast genome by OGDRAW. The gray arrowheads indicate the direction of the genes. LSC, large single copy region; IR, inverted repeat; SSC, small single copy region. The innermost first black ring indicates the chloroplast genome size of *C. barbatus*. The innermost second and third rings indicate GC content and GC skews deviations in chloroplast genome of *C. barbatus*, respectively: GC skew + indicates G > C, and GC skew − indicates G < C. CGView comparison result of thirteen complete chloroplast genomes in Costaceae displayed from innermost fourth color ring to outwards 16th ring in turn: *C. barbatus* OP712648, *C. beckii* OP712653, *C. dubius* OP712651, *C. speciosus* Guangdong OP712649, *C. speciosus* var. *marginatus* OP712652, *C. tonkinensis* Yunnan OP712650, *C. viridis* MK262733, *C. woodsonii* OP712654, *H. speciosa* Guizhou OK641589, *M. uniflorus* OP712655, *H. lacera* ON598391, *H. speciosa* Yunnan ON598392, and *C. tonkinensis* ON598393; chloroplast genome similar and highly divergent locations are represented by continuous and interrupted track lines, respectively. The species in bold are sequenced in this study

In this study, a total of 13 complete chloroplast genomes of 10 species covering three clades in Costaceae were analyzed, including 8 newly sequenced genomes and 5 published ones (Table 1). The 8 sequenced samples produced 5.97 to 12.47 Gb clean reads each after removal of adapters and low-quality reads (Table S1). The 8 complete chloroplast genomes of Costaceae generated in this study were deposited in the GenBank with accession numbers OP712648 to OP712655 (Table 1). All 13 chloroplast genomes exhibited a typical quadripartite structure containing a pair of inverted repeat (IR) regions (27,982 − 29,203 bp), an LSC region (90,802 − 92,189 bp) and an SSC region (18,363 − 20,124 bp) (Fig. 1; Table 1). The full-length variation of Costaceae was about 2.6 kb (genome size: 166,360 − 168,966 bp). The overall guanine-cytosine (GC) content varied slightly, from 36.16 to 36.55% (Table 1). The IR regions accounted for the highest GC content, followed by the LSC region, while the SSC region had the lowest GC content (Table 1). The GC content of the protein-coding gene sequences ranged from 37.57 to 37.76% (Table 1).

**Table 1:** Basic characteristics of thirteen complete chloroplast genomes of the Costaceae family

Species	GenBank accession	Size (bp)	LSC (bp)	SSC (bp)	IR (bp)	GC content (%)					Number of genes (different)	Number of CDS (different)	Number of tRNA (different)	Number of rRNA (different)	Genes with introns
						Total	LSC	SSC	IR	CDS					
*C. barbatus*	OP712648	168,717	91,971	18,414	29,166	36.29	34.11	29.62	41.82	37.57	134 (111)	88 (79)	38 (28)	8 (4)	18
*C. speciosus* Guangdong	OP712649	167,174	91,239	19,971	27,982	36.33	34.28	29.15	42.23/ 42.24	37.61	134 (112)	88 (79)	38 (29)	8 (4)	18
C. *tonkinensis* Yunnan	OP712650	166,360	90,802	19,524	28,017	36.55	34.51	29.69	42.24/ 42.25	37.68	134 (112)	88 (79)	38 (29)	8 (4)	18
*C. dubius*	OP712651	168,573	91,938	18,363	29,136	36.30	34.13	29.63	41.83	37.58	134 (111)	88 (79)	38 (28)	8 (4)	18
*C. speciosus* var. *marginatus*	OP712652	167,185	91,236	19,953	27,998	36.33	34.28	29.17	42.22	37.63	134 (112)	88 (79)	38 (29)	8 (4)	18
*C. beckii*	OP712653	168,719	91,983	18,404	29,166	36.29	34.12	29.64	41.82	37.58	135 (111)	88 (79)	39 (28)	8 (4)	17
*C. woodsonii*	OP712654	168,551	91,861	18,420	29,135	36.30	34.15	29.55	41.83	37.58	134 (111)	88 (79)	38 (28)	8 (4)	18
*M. uniflorus*	OP712655	168,484	91,271	18,807	29,203	36.31	34.27	29.07	41.82/ 41.83	37.61	134 (112)	88 (79)	38 (29)	8 (4)	18
*H. speciosa* Guizhou	OK641589	167,158	91,239	19,955	27,982	36.33	34.28	29.17	42.23/ 42.24	37.60	134 (113)	88 (79)	38 (30)	8 (4)	18
*C. viridis*	MK262733	168,966	92,189	18,445	29,166	36.25	34.06	29.60	41.20	37.76	134 (111)	88 (79)	38 (28)	8 (4)	18
*H. lacera*	ON598391	168,053	91,955	20,124	27,987	36.16	34.03	29.00	42.23	37.70	134 (113)	88 (79)	38 (30)	8 (4)	18
*H. speciosa* Yunnan	ON598392	167,626	91,673	19,989	27,982	36.21	34.08	29.12	42.23	37.68	134 (113)	88 (79)	38 (30)	8 (4)	18
*C. tonkinensis*	ON598393	167,481	91,538	19,881	28,031	36.37	34.32	29.35	42.22	37.72	134 (113)	88 (79)	38 (30)	8 (4)	18

*Note: CDS* protein-coding genes, *GC* guanine-cytosine, *LSC* large single copy region, *SSC* small single copy region, *IR* inverted repeat, OP712648-OP712655 generated in this study

Herein,134 − 135 genes were annotated in these 13 genomes of Costaceae, consisting of 88 protein-coding genes, 8 ribosomal RNA genes (rRNAs) and 38 − 39 transfer RNA genes (tRNAs) (Table 1, Table S2). After annotation and manual checking, individual chloroplast genome resulted in 111 − 113 different genes, comprising 79 different protein-coding genes, 28 − 30 different tRNAs and 4 different rRNAs (Fig. 1; Tables 1 and 2, Table S2). Among all 13 genomes, the numbers of different protein-coding genes and different rRNAs were the same, but slight differences were found in tRNAs (Table 2, Table S2).

**Table 2:** Gene contents in thirteen complete chloroplast genomes of the Costaceae family

Category of genes	Group of genes	Name of genes
Self-replication	DNA dependent RNA polymerase	*rpoA*, *rpoB*, rpoC1*, *rpoC2*
	Large subunit of ribosomal proteins	*rpl2* (×2)*, *rpl14*, rpl16*, *rpl20*, *rpl22*, *rpl23* (×2), *rpl32*, *rpl33*, *rpl36*
	Small subunit of ribosomal proteins	*rps2*, *rps3*, *rps4*, *rps7* (×2), *rps8*, *rps11*, *rps12* (×2)*, *rps14*, *rps15*, rps16*, *rps18*, *rps19* (×2)
RNA genes	Ribosomal RNA	*rrn4.5* (×2), *rrn5* (×2), *rrn16* (×2), *rrn23* (×2)
	Transfer RNA	*trnA-UGC* (×2)*, *trnC-GCA*, *trnD-GUC*, *trnE-UUC*, *trnF-GAA*, *trnG-GCC* (×2)⑥, trnG-UCC*①, *trnH-GUG* (×2), *trnI-GAU* (×2)*, trnK-UUU*, *trnL-CAA* (×2), trnL-UAA*, *trnL-UAG*, *trnM-CAU* (×4), *trnN-GUU* (×2), *trnP-UGG*, *trnQ-UUG*, *trnR-ACG* (×2), *trnR-UCU*, *trnS-GCU*, *trnS-GGA*⑤, *trnS-UGA*⑤, *trnT-GGU*⑤, *trnT-UGU*, *trnV-GAC* (×2), trnV-UAC*, *trnW-CCA*, *trnY-GUA*, *trnfM-CAU*②, *trnI-CAU*③, *trnS-CGA*④
Photosynthesis related genes	Subunits of photosystem I	*psaA*, *psaB*, *psaC*, *psaI*, *psaJ*
	Subunits of photosystem II	*psbA*, *psbB*, *psbC*, *psbD*, *psbE*, *psbF*, *psbH*, *psbI*, *psbJ*, *psbK*, *psbL*, *psbM*, *psbN*, *psbT*, *psbZ*, *infA*
	Subunits of cytochrome b/f complex	*petA*, petB*, petD*, *petG*, *petL*, *petN*
	Subunits of ATP synthase	*atpA*, *atpB*, *atpE*, atpF*, *atpH*, *atpI*
	Subunits of NADH dehydrogenase	ndhA*, *ndhB* (×2)*, *ndhC*, *ndhD*, *ndhE*, *ndhF*, *ndhG*, *ndhH*, *ndhI*, *ndhJ*, *ndhK*
	Subunit of rubisco	*rbcL*
Other genes	Subunit of acetyl-coA-carboxylase	*accD*
	c-type cytochrome synthesis gene	*ccsA*
	Envelop membrane protein	*cemA*
	Protease	clpP**
	Maturase	*matK*
Genes of unknown function	Conserved open reading frames	*ycf1* (×2), *ycf2* (×2), ycf3**, *ycf4*

*Note: * *: gene containing one intron; **: gene containing two introns; (×2): gene with two copies; (×4): gene with four copies; ①: *trnG-UCC* has no intron in chloroplast genome of *C. beckii*; ②: *trnfM-CAU* is missing in four chloroplast genomes of *C. barbatus*, *C. dubius*, *C. beckii* and *C. woodsonii*, respectively; ③: *trnI-CAU* is only present in five chloroplast genomes of *H. speciosa* Guizhou, *C. viridis*, *H. lacera*, *H. speciosa* Yunnan, and *C. tonkinensis*, respectively; ④: *trnS-CGA* is only present in chloroplast genome of *C. viridis*; ⑤: *trnS-GGA*, *trnS-UGA*, and *trnT-GGU* are missing in chloroplast genome of *C. viridis*; ⑥: *trnG-GCC* has two copies only in chloroplast genome of *C. beckii*

Among these 111 − 113 different genes, 21 genes were duplicated within IR regions, including 9 protein-coding genes, 8 tRNAs, and 4 rRNAs (Fig. 1; Table 2, Table S2). Sixteen genes contained one intron, while *clpP* and *ycf3* each contained two introns in 12 chloroplast genomes except in genome of *C. beckii* (Table 2, Table S2). The genome of *C. beckii*, only contained 17 intron-containing genes, because *trnG-UCC* has lost the intron (Table 2, Table S2).

### Long repeats and SSRs analyses

**Fig. 2:**
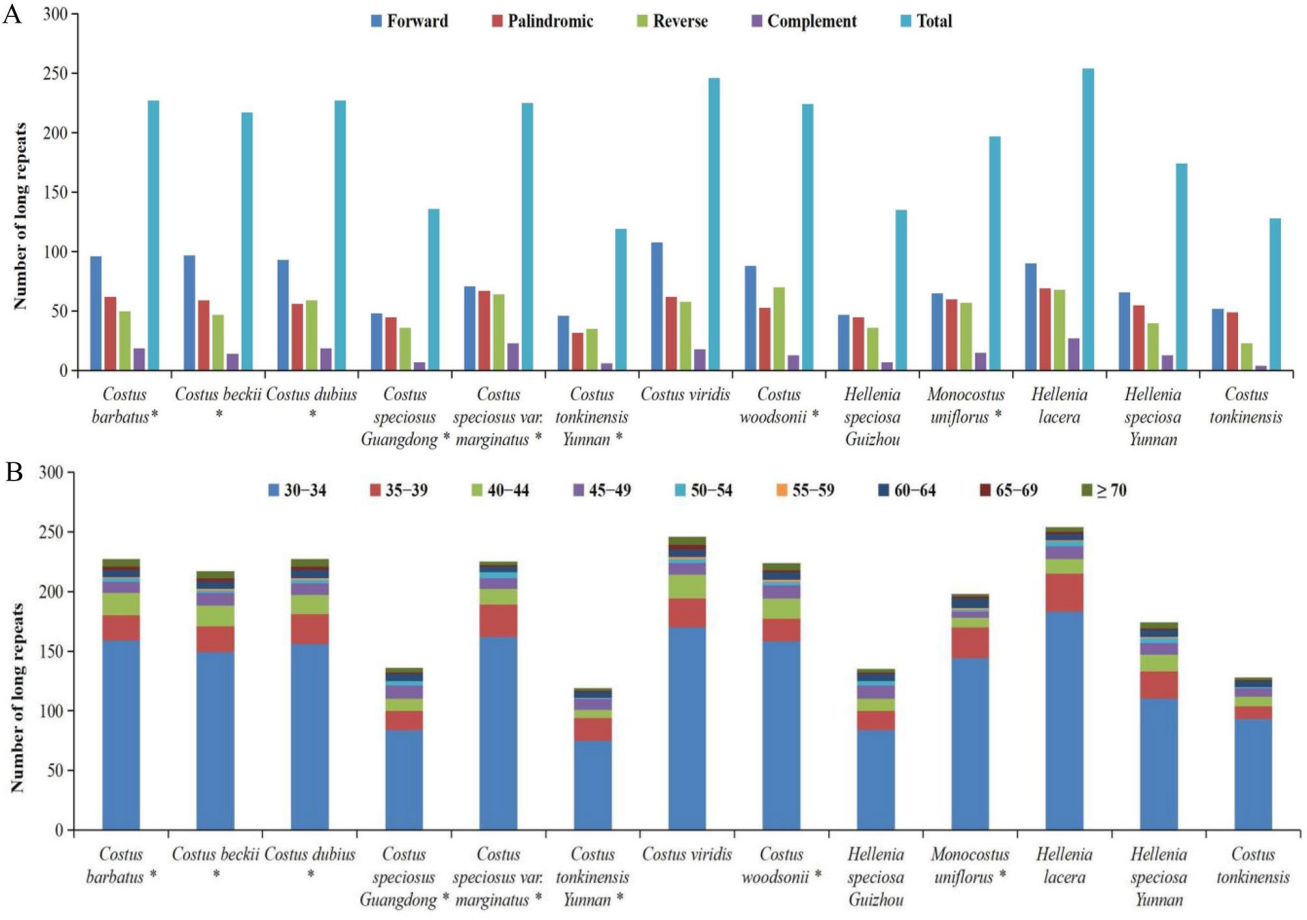
Analysis of long repeats in thirteen complete chloroplast genomes of the Costaceae family. (**A**), Total numbers and different types of long repeats in each chloroplast genome. (**B**), Numbers of long repeats more than 30 bp long in each chloroplast genome. * indicates chloroplast genome of the species sequenced in this study

Four types of long repeats, including forward, complement, reverse and palindromic repeats, were detected in 13 complete chloroplast genomes of Costaceae. Among these 13 genomes, *H. lacera* ON598391 contained the highest number of long repeats (254), and *C. tonkinensis* Yunnan OP712650 contained the lowest number of long repeats (119) (Fig. 2A, Table S3). The number of forward repeats varied from 46 (*C. tonkinensis* Yunnan OP712650) to 108 (*C. viridis* MK262733), the number of palindromic repeats varied from 32 (*C. tonkinensis* Yunnan OP712650) to 69 (*H. lacera* ON598391), the number of reverse repeats varied from 23 (*C. tonkinensis* ON598393) to 70 (*C. woodsonii* OP712654), and the number of complement repeats varied from 4 (*C. tonkinensis* ON598393) to 27 (*H. lacera* ON598391) (Fig. 2A, Table S3). The lengths of the long repeats varied among the 13 genomes, of which most were found to exist with the range of 30 − 34 bp (Fig. 2B, Table S3). Long repeats with lengths of 35 − 39 bp and 40 − 44 bp were the second and third most common, respectively (Fig. 2B, Table S3).

**Fig. 3:**
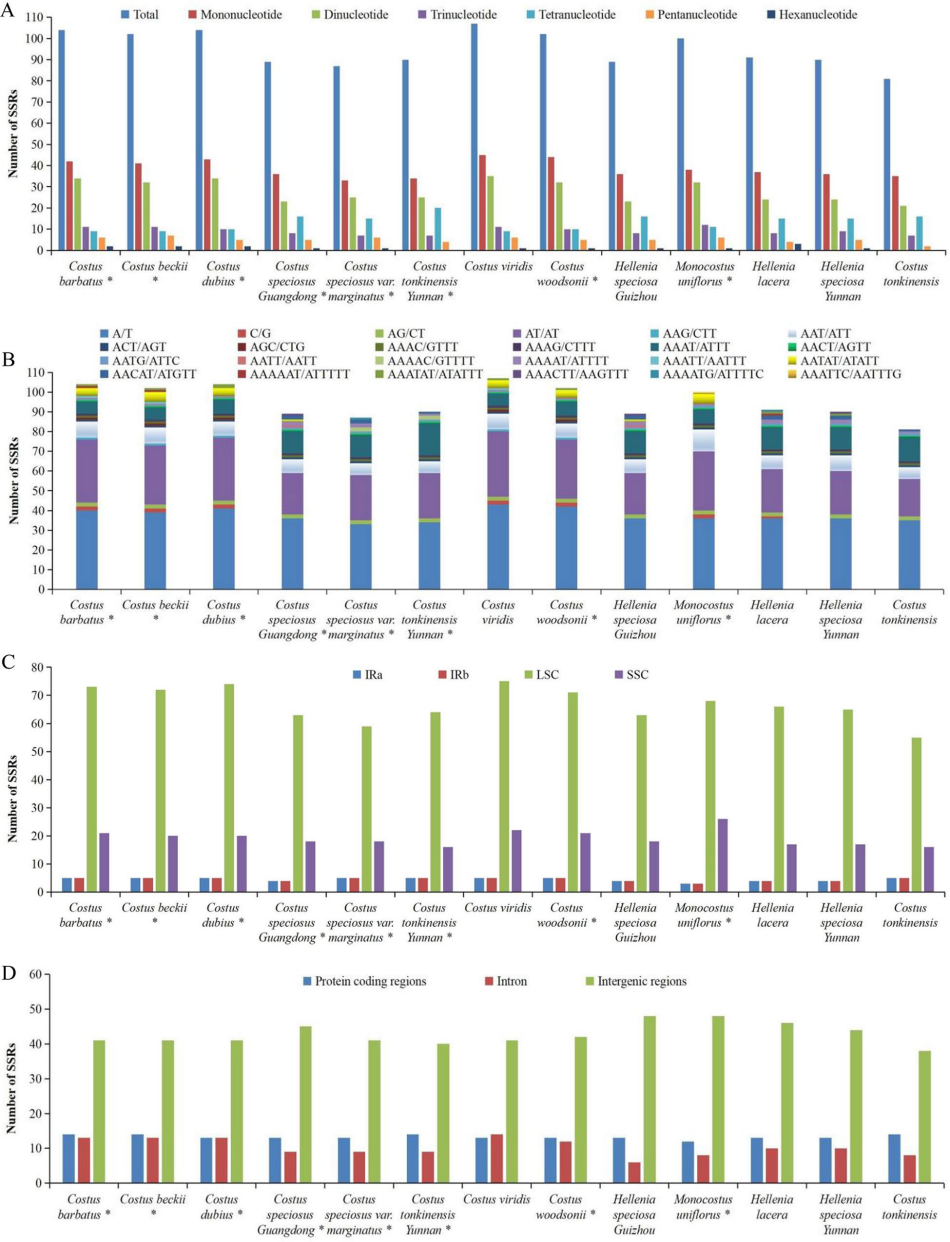
Analysis of SSRs in thirteen complete chloroplast genomes of the Costaceae family. (**A**), Total numbers and different types of SSRs detected in each chloroplast genome. (**B**), Frequencies of the identified SSRs in different motifs. (**C**), Frequencies of the identified SSRs in the LSC, SSC and IR regions. (**D**), SSR distribution in protein-coding regions, introns and intergenic regions detected in each chloroplast genome. * indicates chloroplast genome of the species sequenced in this study

Simple sequence repeats (SSRs) in these 13 complete chloroplast genomes of Costaceae were also detected (Fig. 3, Table S4). The number of SSRs detected among these 13 genomes ranged from 81 (*C. tonkinensis* ON598393) to 107 (*C. viridis* MK262733) (Fig. 3A, Table S4). Among these SSRs, only 2 chloroplast genomes (*C. tonkinensis* Yunnan OP712650 and *C. tonkinensis* ON598393) had no hexanucleotide repeats (Fig. 3A, Table S4). A/T (39.40%) were the most frequently observed repeats, followed by AT/AT (27.34%), AAAT/ATTT (9.87%) and AAT/ATT (7.77%), respectively (Fig. 3B, Table S4). Among the SSRs in these 13 genomes, each genome contained 55 to 75 SSRs in the LSC regions, 16 to 26 SSRs in the SSC regions, and 3 to 5 SSRs in the IRa and IRb regions, respectively (Fig. 3C, Table S4). Similarly, SSRs were analyzed in the protein-coding regions, intron regions and intergenic regions of these 13 genomes, indicating that each genome comprised 38 to 48 SSRs in intergenic regions, 12 to 14 SSRs in protein-coding regions, and 6 to 14 SSRs in introns (Fig. 3D, Table S4). Six genes, namely, *ndhD*, *rpoB*, *rpoC2*, *rps14*, *ycf1* and *ycf2* contained SSRs and their products longer than 150 bp in these 13 genomes, which can be used as potential DNA molecular markers for species identification in Costaceae (Table S4).

### Codon usage analysis

**Fig. 4:**
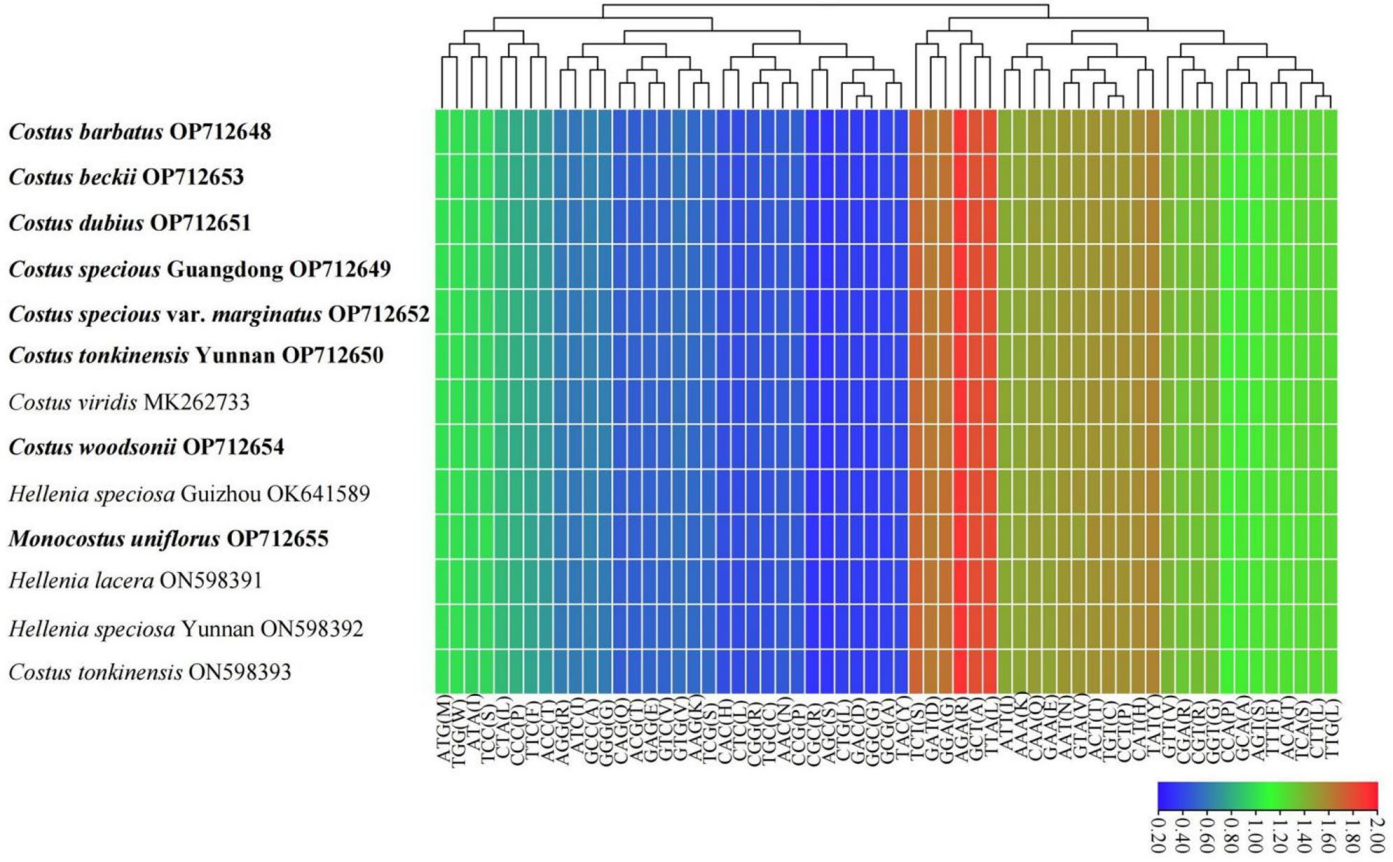
Heat map analysis for relative synonymous codon usage (RSCU) values of all protein-coding genes of thirteen complete chloroplast genomes in the Costaceae family. Red indicates higher RSCU values and blue indicates lower RSCU values. The species in bold are sequenced in this study

The amino acid frequency, codon usage and relative synonymous codon usage (RSCU) were analyzed based on all 79 different protein-coding genes (Table S5). The total codons (excluding stop codons) of these 13 complete chloroplast genomes of Costaceae ranged from 26,531 to 27,373. Among these codons, leucine (Leu) was the most abundant amino acid, followed by isoleucine (Ile); whereas cysteine (Cys) was the least abundant (Table S5). The codons ATG and TGG, encoding methionine (Met) and tryptophan (Trp), respectively, showed no codon bias both with RSCU values of 1.00 in these 13 genomes (Fig. 4, Table S5). The codons with the five lowest RSCU values (AGC, GAC, GGC, CTG and CGC) and three with the highest RSCU values (AGA, GCT, and TTA) were found in these 13 genomes (Fig. 4, Table S5). Twenty-nine codons showed codon usage bias with RSCU > 1.00 in these 13 genomes genes (Table S5). Interestingly, of these 29 codons, twenty-eight were A/T-ending codons. The result of higher usage frequency of A/T-ending than G/C-ending was also found in *Aglaonema modestum* [29], *Phaseolus lunatus* [32], and *Zingiber montanum* [33].

### IR expansion and contraction

**Fig. 5:**
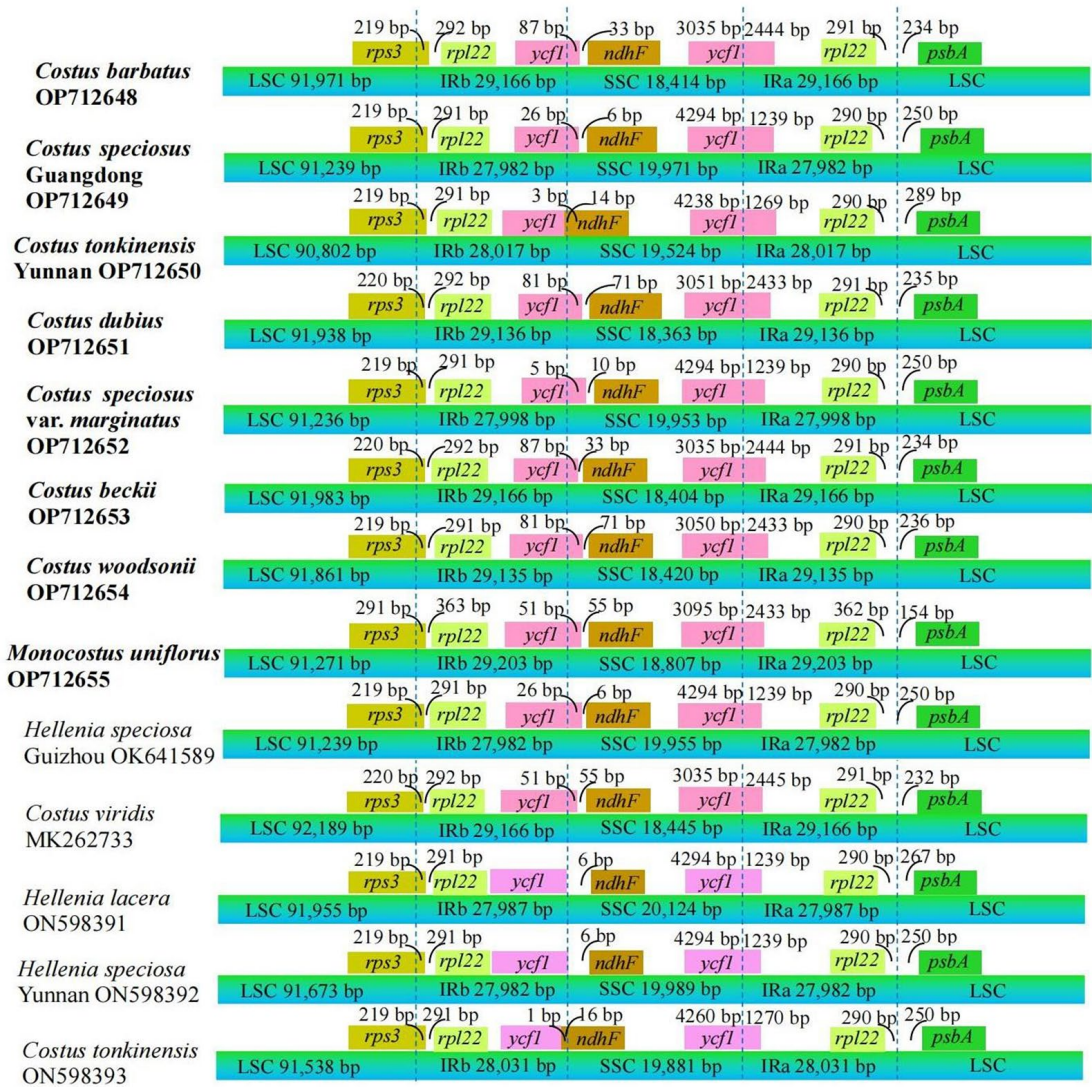
Comparisons of border distances between adjacent genes and junctions of the LSC, SSC and two IR regions among thirteen complete chloroplast genomes of the Costaceae family. Numbers above or near the colored genes indicate the distances between the genes and the boundary sites. The figure is not in scale for sequence length, and only shows relative changes at or near the IR/SC boundaries. The species in bold are sequenced in this study

Detail comparisons at the LSC/IRs/SSC boundaries were analyzed among the 13 complete chloroplast genomes of Costaceae (Fig. 5). Although the IR/LSC boundaries of these 13 genomes were highly conserved, variations were also found in the IR/SSC boundaries. For IRa/LSC boundaries, the *rpl22* and *psbA* genes were located at the boundaries in these 13 genomes, respectively. The distances between the ends of *rpl22* and IRa/LSC boundaries ranged from 290 to 362 bp, and the distances between the starts of *psbA* and the IRa/LSC boundaries ranged from 154 to 289 bp (Fig. 5). Among these 13 genomes, the *rps3* and *rpl22* genes were found at the boundaries of the LSC/IRb regions, respectively (Fig. 5). *rps3* expanded into the IRb regions in these 13 genomes, with the lengths ranging from 219 to 291 bp from the LSC/IRb boundaries; whereas the starts of *rpl22* and the LSC/IRb boundaries ranged from 291 to 363 bp (Fig. 5).

For SSC/IRa boundaries, *ycf1* was located in the boundaries in these 13 genomes, which crossed into the IRa regions with lengths varying from 1239 to 2445 bp (Fig. 5). Regarding the IRb/SSC boundaries, *ycf1* and *ndhF* genes were located at the boundaries in these 13 genomes, respectively (Fig. 5). *ycf1* expanded into the SSC regions ranging from 3 to 87 bp in 10 genomes, respectively (Fig. 5). In contrast, the end of the *ycf1* gene was justly located within the IRb/SSC boundaries in 2 genomes (*H. lacera* and *H. speciosa* Yunnan) (Fig. 5). In the rest of the genome (*C. tonkinensis* ON598393), the distance between the end of *ycf1* and the IRb/SSC boundary was 1 bp (Fig. 5). Among the 11 genomes, the lengths between the starts of *ndhF* and the IRb/SSC boundaries ranged from 6 to 71 bp, respectively (Fig. 5). However, in the other 2 genomes (*C. tonkinensis* Yunnan OP712650 and *C. tonkinensis* ON598393), *ndhF* expanded into the IRb regions by 14 and 16 bp, respectively (Fig. 5).

### Sequence divergence analysis and nucleotide diversity

**Fig. 6:**
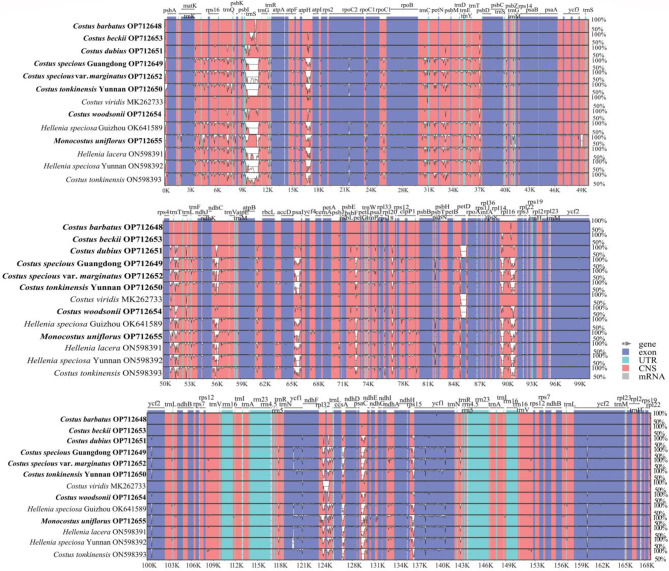
Visualized alignment of thirteen complete chloroplast genomes sequences of the Costaceae family using mVISTA. *C. barbatus* chloroplast genome sequence was used as a reference. Gray arrows and thick black lines indicate gene orientation. Purple bars represent exons, sky-blue bars represent untranslated regions (UTRs), red bars represent non-coding sequences (CNS), gray bars represent mRNA and white regions represent sequence differences among all analyzed chloroplast genomes. Horizontal axis indicates the coordinates within the chloroplast genome. Vertical scale represents the identity percentage that ranges from 50–100%. The species in bold are sequenced in this study

Using the whole chloroplast genome of *C. barbatus* as the reference, a comparative analysis based on the mVISTA program was performed on the 13 complete chloroplast genomes of Costaceae (Fig. 6). The results indicated that the LSC and SSC regions were more divergent than the two IR regions (Fig. 6). In the protein-coding regions, most protein-coding genes were highly conserved except for *rpl16*, *rpoC1*, *ccsA*, *ndhF*, *psaJ*, *rps3*, *rps15* and *ycf1* (Fig. 6). The highly divergent regions among these 13 genomes mainly located in the intergenic regions, including *trnS*-*trnG*, *atpH*-*atpI*, *accD*-*psaI* and *rpl16-exon2*-*rpl16-exon1* in the LSC region as well as *ndhF-rpl32*, *rpl32*-*trnL*, *ccsA-ndhD*, *psaC-ndhE* and *rps15*-*ycf1* in the SSC region (Fig. 6). The CGview result also revealed that the IR regions were less divergent than the LSC and SSC regions (innermost 4th color ring to the outwards 16th ring in Fig. 1). In comparison to the chloroplast genome of *C. barbatus* (innermost 4th color ring in Fig. 1), the rest of the 12 genomes showed four divergent regions in LSC (*psbI-trnS*, *trnS-trnG*, *trnT-trnE*, and *rps3*), one region in SSC (*ccsA-ndhD*) and one region in IRa (*rpl22-rps19*).

**Fig. 7:**
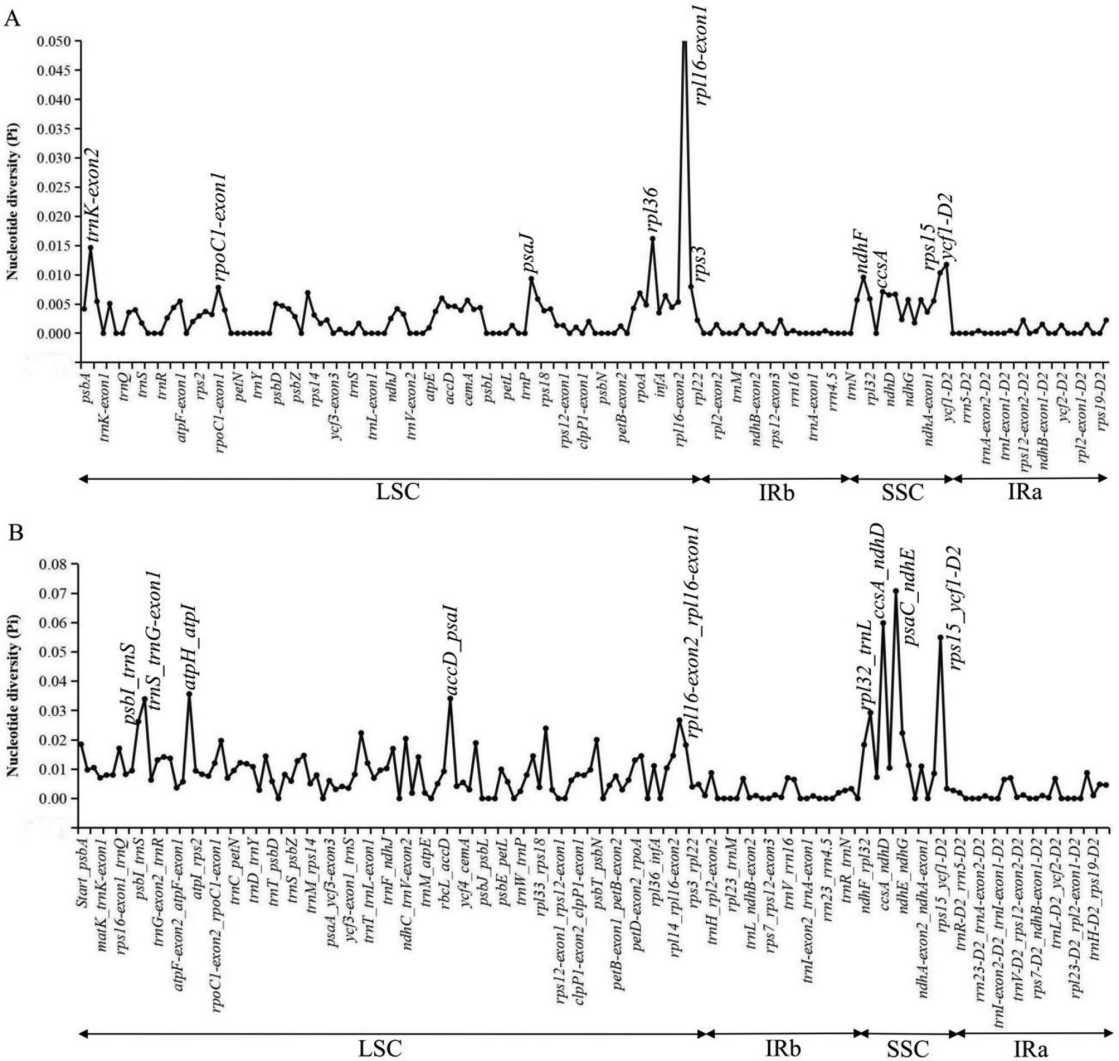
Comparisons of nucleotide diversity (Pi) values among thirteen complete chloroplast genomes of the Costaceae family. (**A**), Protein-coding genes. Protein-coding genes with Pi values > 0.007 are labeled with gene names. (**B**), Intergenic regions. Intergenic regions with Pi values > 0.025 are labeled with intergenic region names

Nucleotide diversity (Pi) and single nucleotide substitutions in the LSC, SSC, IRa, IRb and the total of the chloroplast genomes were analyzed (Table 3). Thirteen complete chloroplast genomes of Costaceae were aligned with a matrix of 168,717 bp with 3,161 variable sites (1.87%) and 3,070 parsimony informative sites (1.82%). The Pi value of the complete chloroplast genome was 0.006 (Table 3). The SSC region had the highest Pi value (0.015) and the IRb region had the lowest Pi value (0.001) (Table 3). Additionally, Pi values were measured by DnaSP v. 6.12.03 to identify highly variable regions in these 13 genomes (Fig. 7, Table S6). Of the protein-coding regions, the Pi value for each gene ranged from 0 to 0.0598, and the average value was 0.0026. The *rpl16-exon1* had the highest Pi value (0.0598) followed by the other nine gene regions of *rpl36*, *trnK-exon2*, *ycf1-D2*, *rps15*, *ndhF*, *psaJ*, *rps3*, *rpoC1-exon1* and *ccsA* (Pi > 0.007) (Fig. 7A, Table S6). For the intergenic regions, the Pi values ranged from 0 to 0.0708 (*psaC-ndhE*) and had an average of 0.0081. The average Pi value of intergenic regions was 3.11 folds higher than that in protein-coding regions. Nine of these intergenic regions also showed remarkably high values (Pi > 0.025), including *psaC-ndhE*, *ccsA-ndhD*, *rps15-ycf1-D2*, *atpH-atpI*, *accD-psaI*, *trnS-trnG-exon1*, *rpl32-trnL*, *rpl16-exon2-rpl16-exon1* and *psbI-trnS* (Fig. 7B). Four universal chloroplast DNA markers, namely, *trnL-F* locus (*trnL-exon2*-*trnF*), *trnL* intron (*trnL-exon1*-*trnL-exon2*), *trnK* locus (*matK*-*trnK-exon1*) and *trnK*-*rps16* inergenic spacer (*trnK-exon1*-*rps16-exon2*) were also tested on their variability. These four chloroplast DNA markers had Pi values of 0.0096, 0.0069, 0.0070 and 0.0079, respectively (Table S6). The Pi values of these four DNA markers were much lower than those of the newly identified highly variable intergenic regions.

**Table 3:** Variable site analyses of thirteen complete chloroplast genomes of the Costaceae family

Regions	Length	Variable sites		Informative sites		Nucleotide diversity
		Number	%	Number	%	
LSC	91,971	2,028	2.2050	1,967	2.1387	0.0075
SSC	18,414	839	4.5563	814	4.4205	0.0154
IRa	29,166	180	0.6171	170	0.5829	0.0020
IRb	29,166	114	0.3909	112	0.3840	0.0013
Complete chloroplast genome	168,717	3,161	1.8736	3,070	1.8196	0.0063

**Fig. 8:**
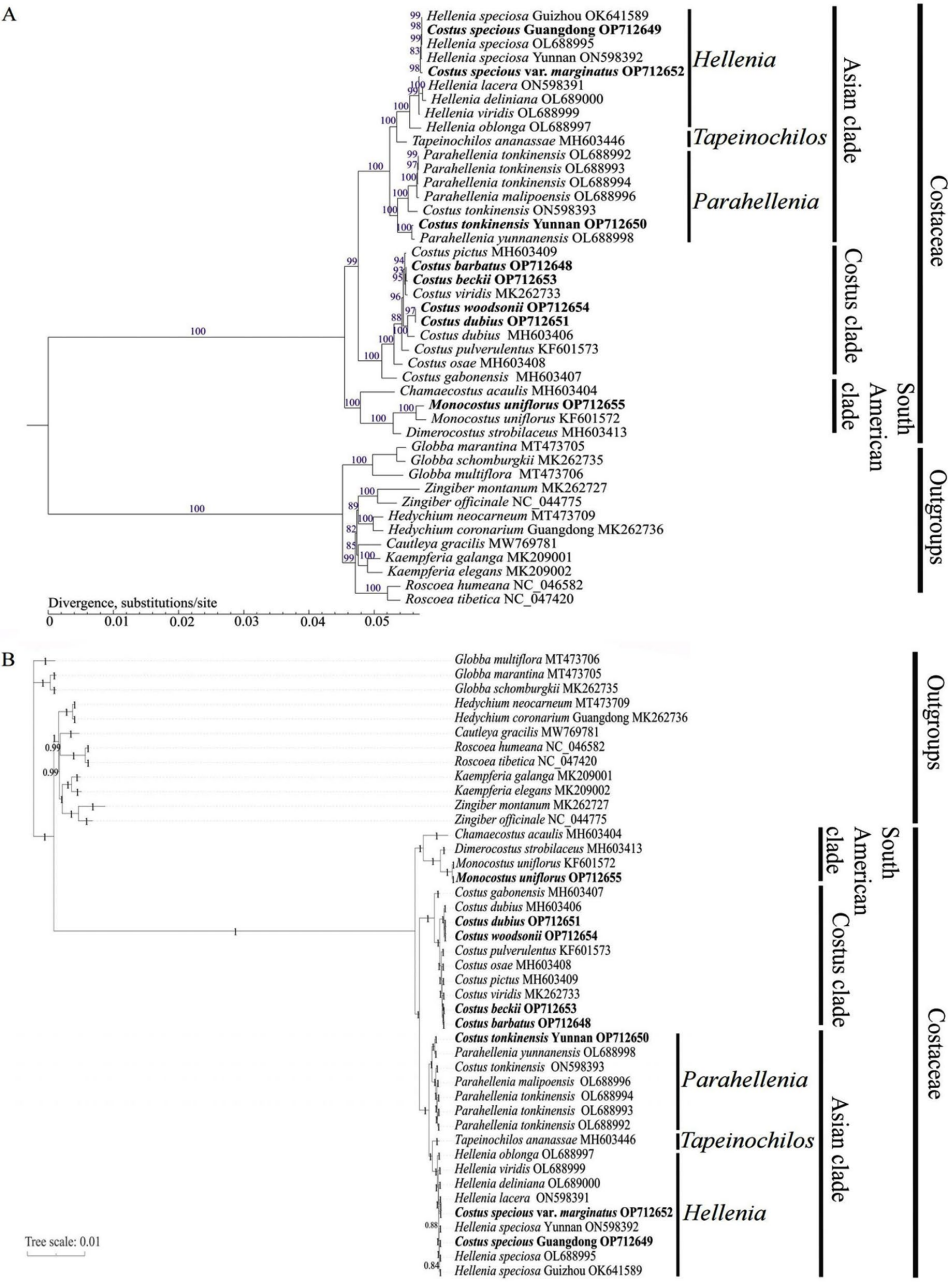
Phylogenetic relationships of Costaceae species based on chloroplast genomes sequences reconstructed using maximum likelihood (ML) and the bayes inference (BI) methods. (**A**), ML tree. (**B**), BI tree. The species in bold are sequenced in this study

By using region length > 250 bp and integrating the results of Pi, CGView and mVISTA, 18 regions, including 14 divergent regions and 4 universal chloroplast DNA markers, were extracted and constructed using the maximum likelihood (ML) trees to differentiate these 13 species/accessions of Costaceae (Additional file 7, Fig. S1). The basic topological structures of the ML trees, which were consistent with topological structures constructed by chloroplast genome data (Fig. 8), were selected for resolution power analysis. The resolution power depended on the number of discrimination successes in the ML trees. If the bootstrap value of the node between two species/accessions was more than 50, species/accessions in the ML tree were counted. Otherwise, species/accessions in the ML tree were not counted. The ML trees constructed by five divergent regions (*ndhF, ycf1-D2*, *ccsA-ndhD*, *rps15-ycf1-D2* and *rpl16-exon2-rpl16-exon1*), and four universal chloroplast DNA markers (Fig. S1), were consistent with topological structures constructed by chloroplast genome data (Fig. 8). The four universal chloroplast DNA markers had resolution powers of *trnL-exon1*-*trnL-exon2* at 46%, *trnK-exon1*-*rps16-exon2* at 31%, *matK*-*trnK-exon1* at 15% and *trnL-exon2*-*trnF* at 0, respectively (Fig. S1a, b, c, d). Comparative analysis of these five potential new markers revealed that *ycf1-D2* had the highest resolution power of 69%, followed by *ndhF* at 46%, *rpl16-exon2-rpl16-exon1* at 38%, *ccsA-ndhD* at 31%, and *rps15-ycf1-D2* at 31% (Fig. S1f, i, l, m, r). Single candidate new marker with differentiation success of 100% was not found. These five regions (*ndhF, ycf1-D2*, *ccsA-ndhD*, *rps15-ycf1-D2* and *rpl16-exon2-rpl16-exon1*) were combined as new potential markers. These five combined potential markers (*ycf1-D2* + *ndhF*, *ccsA-ndhD* + *rps15-ycf1-D2*, *ccsA-ndhD* + *rpl16-exon2-rpl16-exon1*, *rps15-ycf1-D2* + *rpl16-exon2-rpl16-exon1*, and *ccsA-ndhD* + *rps15-ycf1-D2* + *rpl16-exon2-rpl16-exon1*) showed differentiation success ≧ 69%, especially, the ML tree constructed from *ccsA-ndhD* + *rps15-ycf1-D2* with high supports (bootstrap values > 65%, and resolution power at 92%), could be used as a candidate molecular marker in Costaceae (Fig. S1s, t, u, v, w).

### Selective pressure analysis

The ratio (ω) of non-synonymous (dN) to synonymous (dS) substitution (dN/dS) for all 79 shared protein-coding genes was analyzed across 13 complete chloroplast genomes in Costaceae. According to the M8 (β & ω > 1) model, a total of 8 protein-coding genes were under positive selection with posterior probability greater than 0.95 using the Bayes empirical bayes (BEB) method (Table 4). Among these genes, *ndhA* harboured the highest number of positive amino acids sites (6), followed by *rps12* (3), *ycf1* (3), *clpP* (2), *petB* (2), *psbD* (2), *cemA* (1) and *ndhF* (1) (Table 4). However, the M2a model analysis revealed that there were only 14 positive amino acid sites by using the BEB method (Table 4). These results inferred that the M8 model was significantly better than the M2a model, identifying the presence of amino acid sites under positive selection.

**Table 4:** Positively selected sites detected in thirteen complete chloroplast genomes of the Costaceae family

Gene name	Model	np	lnL	Parameters	Positively selected sites Pr (ω > 1)
*cemA*	M2a	29	-991.852756	ω2 = 16.05889	1 M 0.931
	M8	29	-991.966607	p0 = 0.95614 *p* = 0.00500 q = 2.03556 (p1 = 0.04386) ω = 10.52669	1 M 0.963*
*clpP*	M2a	29	-844.212769	ω2 = 145.50270	23 L 0.919, 24I 0.998**
	M8	29	-844.212761	p0 = 0.98974 *p* = 0.00500 q = 1.93438 (p1 = 0.01026) ω = 145.50213	23 L 0.958*, 24I 0.999**
*ndhA*	M2a	29	-1624.988245	ω2 = 102.62253	83 V 0.933, 185R 1.000**, 186 V 1.000**, 187I 1.000**, 188 L 1.000**, 200 W 0.962*
	M8	29	-1637.945062	p0 = 0.95909 *p* = 0.00500 q = 1.93014 (p1 = 0.04091) ω = 30.35637	83 V 0.963*, 185R 1.000**, 186 V 1.000**, 187I 1.000**, 188R 1.000**, 200 W 0.981*
*ndhF*	M2a	29	-3304.560615	ω2 = 4.42852	674 A 0.963*
	M8	29	-3304.561625	p0 = 0.96774 *p* = 0.52675 q = 8.62704 (p1 = 0.03226) ω = 4.45145	674 A 0.971*
*petB*	M2a	29	-905.857200	ω2 = 375.89354	1 L 1.000**, 2 N 0.991**
	M8	29	-907.599426	p0 = 0.98507 *p* = 0.00500 q = 2.19711 (p1 = 0.01493) ω = 208.93829	1 L 1.000**, 2 N 0.996**
*psbD*	M2a	29	-1489.475590	ω2 = 179.56481	3I 0.996**, 4 A 1.000**
	M8	29	-1489.475572	p0 = 0.99133 *p* = 0.00500 q = 1.97892 (p1 = 0.00867) ω = 179.56381	3I 0.998**, 4 A 1.000**
*rps12*	M2a	29	-518.619174	ω2 = 999.00000	18R 0.932, 55Q 0.932, 115 K 0.991**
	M8	29	-518.619174	p0 = 0.94820 *p* = 0.00500 q = 1.93325 (p1 = 0.05180) ω = 999.00000	18R 0.976*, 55Q 0.977*, 115 K 0.998**
*ycf1*	M2a	29	-8514.166872	ω2 = 6.86864	916 K 0.944, 1130I 0.961*, 1416 K 0.985*
	M8	29	-8514.114591	p0 = 0.97844 *p* = 0.30668 q = 0.74177 (p1 = 0.02156) ω = 7.56416	916 K 0.969*, 1130I 0.978*, 1416 K 0.994**

*Note:* * and ** indicate posterior probability higher than 0.95 and 0.99, respectively

### Phylogenetic relationships

Two phylogenetic trees were constructed using chloroplast genome sequences by ML and Bayes inference (BI) methods, respectively (Fig. 8A and B). The species of Zingiberaceae were used as outgroups. Both ML and BI trees displayed similar topological structures (Fig. 8A and B). The analyzed Costaceae species were divided into three clades: a South American clade, an Asian clade and a *Costus* clade with strongly supported values (bootstrap values = 99–100% for the ML tree and posterior probabilities = 1 for the BI tree nodes) (Fig. 8A and B).

In both two trees, there were three subclades in the Asian clade with strong supports (bootstrap values = 100%; posterior probabilities = 1), namely, *Hellenia*, *Tapeinochilos* and *Parahellenia*, which had nested relationships (Fig. 8A and B). Within *Hellenia*, *H. speciosa* Guizhou OK641589, *C. speciosus* Guangdong OP712649, *H. speciosa* OL688995, *H. speciosa* Yunnan ON598392 and *C. speciosus* var. *marginatus* OP712652 were clustered one by one, forming a cluster with moderate to strong supports (bootstrap values = 83 − 100%; posterior probabilities = 0.84 − 1); *H. lacera* ON598391 and *H. delinana* OL689000 were clustered together, forming another cluster with strong supports (bootstrap value = 100%; posterior probability = 1); then the two clusters, *H. viridis* OL688999 and *H. oblonga* OL688997 were clustered step by step (Fig. 8A and B). Within *Parahellenia*, three accessions of *P. tonkinensis* (OL688992, OL688993 and OL688994), *P. malipoensis* OL688996 and *C. tonkinensis* ON598393 were clustered together, forming a cluster with strong supports (bootstrap values = 97 − 100%; posterior probabilities = 1); *C. tonkinensis* Yunnan OP712650 and *P. yunanensis* OL688998 were clustered together, forming another cluster with strong supports (bootstrap value = 100%; posterior probability = 1.0); then the two clusters were clustered together with strong supports (bootstrap value = 100%; posterior probability = 1.0) (Fig. 8A and B). In the *Costus* clade, *C. pictus* MH603409, *C. barbatus* OP712648, *C. beckii* OP712653 and *C. viridis* MK262733 were clustered together, forming a cluster with strong supports (bootstrap value = 93 − 95%; posterior probabilities = 1); *C. woodsonii* OP712654, *C. dubius* OP712651 and *C. dubius* MH603406 were also clustered together, forming another cluster with strong supports (bootstrap value = 97 − 100%; posterior probability = 1); then the two clusters, *C. pulverulentus* KF601573, *C. osae* MH603408 and *C. gabonensis* MH603407 were clustered one by one (Fig. 8A and B). In the South American clade, *M. uniflorus* OP712655 and *M. uniflorus* KF601572 were first clustered together with strong supports (bootstrap value = 100%; posterior probability = 1), then clustered with *Dimerocostus strobilaceus* MH603413 with strong supports (bootstrap value = 100%; posterior probability = 1), and finally clustered with *Chamaecostus acaulis* MH603404 with strong supports (bootstrap value = 100%; posterior probability = 1) (Fig. 8A and B).

### Divergence time estimation

**Fig. 9:**
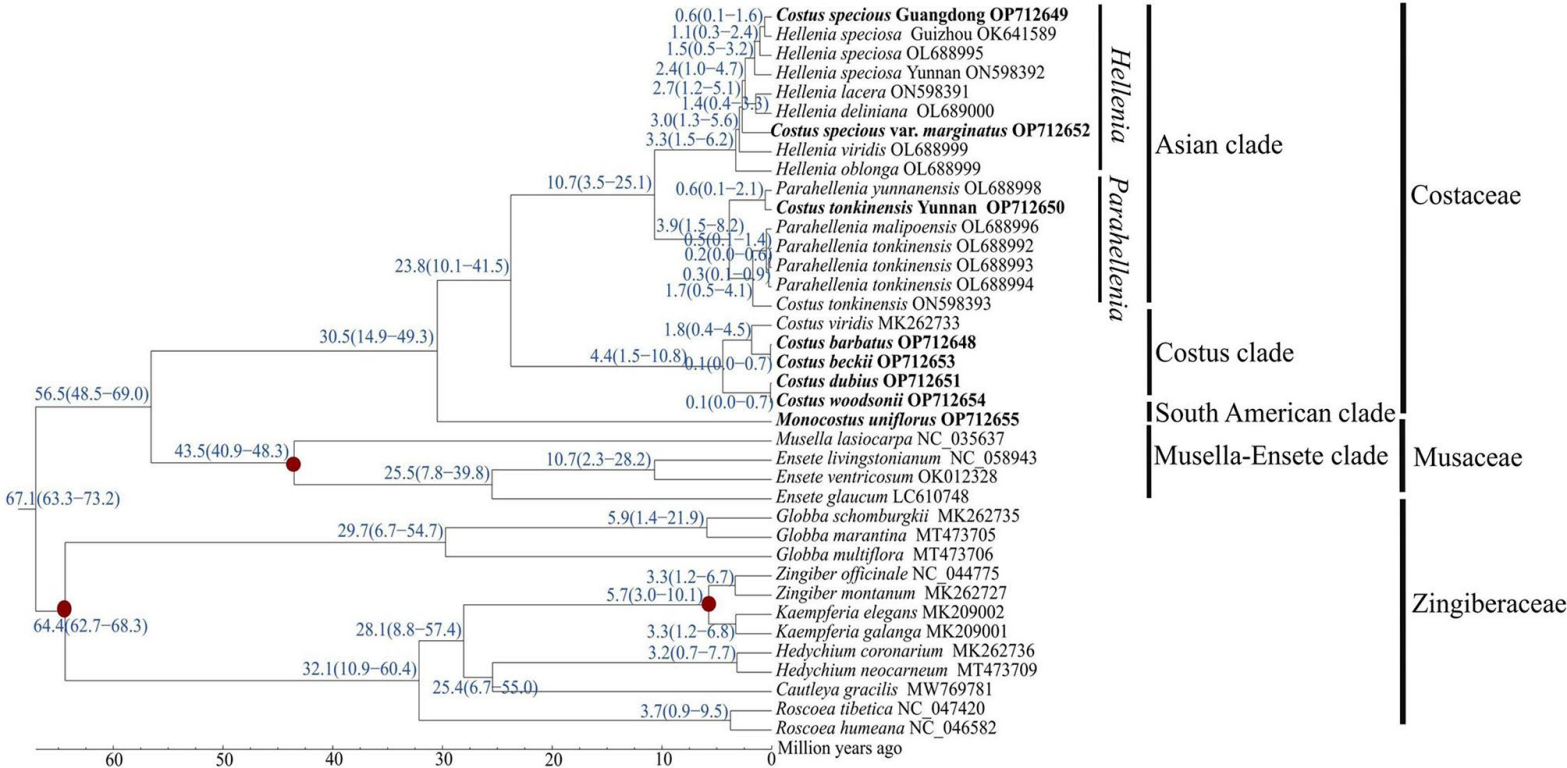
Divergence time estimation of Costaceae species based on nucleotide sequences of 75 single-copy protein-coding genes shared in 22 chloroplast genomes of Costaceae. The fossil and calibration taxa are indicated with red points on the corresponding nodes. Mean divergence time of the nodes are shown at the nodes with blue. The numbers inside each blue bracket after mean divergence time represent 95% highest posterior density (HPD) of estimated divergence time, with minimum and maximum values, respectively. The species in bold are sequenced in this study

Divergence time estimation suggested that the common ancestor of Costaceae firstly split from Zingiberaceae at about 67.1 Mya (95% HPD: 63.3 − 73.2 Mya), and then split from *Musella*-*Ensete* clade at approximately 56.5 Mya (95% HPD: 48.5 − 69.0 Mya) (Fig. 9). The crown node age of Costaceae was about 30.5 Mya (95% HPD: 14.9 − 49.3 Mya) (Fig. 9). The crown node age of the *Costus* clade and Asian clade was 23.8 Mya (95% HPD: 10.1 − 41.5 Mya). Diversification of the *Costus* clade and Asian clade occurred at 4.4 Mya (95% HPD: 1.5 − 10.8 Mya) and 10.7 Mya (95% HPD: 3.5 − 25.1 Mya), respectively. Within the Asian clade, diversification of *Parahellenia* and *Hellenia* took place at 3.9 Mya (95% HPD: 1.5 − 8.2 Mya) and 3.3 Mya (95% HPD: 1.5 − 6.2 Mya), respectively (Fig. 9).

## Discussion

### Chloroplast genome structure and sequence variation

In this study, 13 complete chloroplast genomes of Costaceae were comparatively analyzed. These 13 genomes revealed a typical quadripartite structure, with a single LSC region, a single SSC region and two IR regions (Fig. 1). They shared similar GC content, protein-coding genes, rRNAs and most of the tRNAs, which also had been found in other flowering plants [24–26, 28–35]. Although these 13 genomes were highly conserved, intron loss, gene duplication and gene loss appeared in this study, for instance, *trnG-UCC* had no intron in the genome of *C. beckii*, while the rest 12 genomes contained one intron in this tRNA gene, suggesting that intron loss had occurred during the evolutionary history of *C. beckii*. Interestingly, the genome of *C. viridis* had two copies of *trnG-GCC*, but this tRNA gene showed only one copy in the rest of the 12 genomes (Table 2, Table S2). By contrast, certain events of intron loss, gene duplication and gene loss were reported in Zingiberoideae species [28], *Amorphophallus* species [38] and *Aglaonema* cultivars [29].

IR contraction and expansion have been considered important evolutionary events in chloroplast genomes in some plants, such as genome size variation, gene duplication, and reduction of duplicate genes to one copy [23–25, 29, 39]. Our results also indicated that genome lengths and boundaries of IR expansion existed variations among these 13 genomes. In previous studies, lengths of chloroplast genomes within a genus showed small variations, such as in genera *Ensete* [40] and *Hedychium* [28]. However, the chloroplast genomes of different Costaceae species remarkably varied in genomes lengths of 2.6 kb in this study (Table 1). This occurrence was also reported in the Musaceae species with approximately 5.7 kb differences in genome lengths [40], and the *Polystachya* species with about 3.8 kb differences in genome lengths [31]. The reasons for length variations of chloroplast genomes may be due to the massive number of genes or introns loss and gain, IR contraction and expansion, and variations of the intergenic regions. Although the IR boundaries of these 13 genomes were relatively stable, IR expansion was still observed, such as *rps3* expanding into the LSC/IRb boundaries in all 13 genomes, and *ndhF* expanding into SSC/IRb boundaries in two chloroplast genomes (*C. tonkinensis* Yunnan OP712650 and *C. tonkinensis* ON598393) (Fig. 5). Therefore, variations in LSC/IRb and SSC/IRb boundaries may be the main contributions of IR contraction and expansion in these 13 genomes. The existence of IR contraction and expansion were also reported in previous studies [23–25, 29, 40].

### Highly divergent regions and selective pressure analysis

In previous studies, four universal chloroplast DNA markers, namely, *trnL-F*, *trnL* intron, *trnK* including the *matK* coding region and *trnK*-*rps16* intergenic spacer, had been extensively used for molecular phylogeny and evolution of Costaceae [2, 3, 5, 13]. However, for some *Costus* species, their phylogenetic relationships were poorly resolved by these four chloroplast DNA markers [2, 13]. In the present study, Pi values of these four chloroplast DNA markers were relatively low (Pi < 0.01) compared to other highly divergent regions (Fig. 7, Table S6), which could explain the low-resolution branches found in these phylogenetic studies [2, 13]. Therefore, it is necessary to develop highly variable regions at the family level as potential markers for future research. Here, based on the results of CGview, mVISTA, Pi values and ML trees, 5 highly divergent regions (*ndhF, ycf1-D2*, *ccsA-ndhD*, *rps15-ycf1-D2* and *rpl16-exon2-rpl16-exon1*) among 13 complete genomes of Costaceae were detected, and suitable for species identification (Fig. S1). Similarly, *ccsA-ndhD*, *rps15-ycf1-D2*, *ycf1-D2*, and *ndhF* had been reported for potential molecular markers in Zingiberoideae [28], aroideae [30], *Polystachya* [31] and *Zingiber* [33]. Therefore, the divergent region of *rpl16-exon2-rpl16-exon1* could potentially be used as a specific DNA barcode for species identification and phylogenetic studies in Costaceae. Additionally, to increase the differentiation success of these five divergent regions, five combined regions showed better differentiation power (Fig. S1). Hence, we recommend these five combined regions to be candidate molecular markers to identify Costaceae species.

The ratio (ω = dN/dS) has been widely used for measuring selective pressure [30–31, 33–35]. The ω ratio > 1 represents positive selection, while ω < 1 represents purifying selection [31, 32]. In the current study, the ω ratio was less than one in most of the protein-coding genes, revealing that they were under purifying selection. In addition, 8 genes, namely, *cemA*, *clpP*, *ndhA*, *ndhF*, *petB*, *psbD*, *rps12* and *ycf1*, with positive selection sites were identified in Costaceae in this study (Table 4). Among these genes, two of them (*ndhA* and *ndhF*) encode subunits of NADH-plastoquinone oxidoreductase (Table 2). *ndhA* plays a critical role in the incorporation of the peripheral arm into the membrane-embedded part of the chloroplast NADH dehydrogenase-like (NDH) complex and is essential for stabilizing subcomplex A and subcomplex E of chloroplast NDH complex, which mediates ferredoxin-dependent plastoquinone reduction in the thylakoid membrane [41]. Our results revealed that *ndhA* gene harboured the highest number (6) of positive amino acid sites within the 13 genomes of Costaceae, suggesting that *ndhA* gene may play essential roles in the evolution of chloroplast NDH complex and thylakoid membrane in Costaceae species. The *rps12* encodes ribosome subunit protein and has important effects on the rates and patterns of evolution [27]. The *petB* encodes S1-domain-containing protein of photosynthetic electron transfer B, which is involved in the stabilization and translation of chloroplast mRNAs [42]. Its transcript accumulation is driven by a free-running circadian clock [42]. The *psbD* encodes the core protein D2 of the photosynthesis complex PSII, which is an important factor affecting photosynthetic efficiency during salt stress [43]. The *clpP* encodes caseinolytic protease (Clp) complex, which plays essential roles in maintaining protein homeostasis and comprises both plastid-encoded and nuclear-encoded subunits [44]. Rapid *clpP* sequence evolution is associated with genetic incompatibilities [45]. The *cemA* encodes envelop membrane protein. Lastly, *ycf1* encodes unknown proteins and is competent in identification at genus and species level of orchids [46]. Recent studies have revealed that these eight genes with positive selection in flowering plants are common [27–31, 33, 35, 47, 48]. For example, *ndhF* has been reported as a positive selection in the Aroideae species [30]; *clpP* and *ycf1* have been reported as positive selections in the *Polystachya* species [31]; *ndhA* and *clpP* have been reported as positive selections in the *Hoya* species [35]; *ndhA*, *clpP*, *rps12* and *ycf1* have been reported as positive selections in the *Zingiber* species [33, 47]; and *cemA*, *clpP*, *ndhF*, *petB*, *rps12* and *ycf1* have been reported as positive selections in the *Dalbergia* species [48]. Among the analyzed species of Costaceae, they possessed diversity of ecological habitats, such as shade under the woods, forest margins, moist places in valleys, roadsides and ditch sides [1, 7]. Therefore, Costaceae species may face different types of stresses in their ecological habitats, and these eight positive selection genes may play important roles during the evolution and adaption of the Costaceae species to their respective ecological habitats.

### Phylogenetic analyses and divergence time estimation

Previous reports had used nuclear *ITS* and several chloroplast markers for phylogenetic studies in the Costaceae family [2, 5, 16, 20]. Specifically, two chloroplast DNA markers (*trnK* intron and *trnL-F* spacer) had been extensively used in phylogenetic relationships of Costaceae [2, 5, 16, 20]. These studies based on nuclear *ITS* and two chloroplast markers identified three clades within the Costaceae family: a South American clade, an Asian clade and a *Costus* clade [2, 5, 16, 20]. However, these analyses of phylogenetic relationships in Costaceae contained multiple poor-resolution branches [2, 5, 16, 20]. In this study, both phylogenetic trees obtained by chloroplast genome sequences divided Costaceae into three clades (an Asian clade, a *Costus* clade and a South American clade) with strong support (Fig. 8). Our phylogenetic result was broadly consistent with previous studies [2, 5, 16, 20]. In the Asian clade, three subclades of *Hellenia*, *Tapeinochilos* and *Parahellenia* displayed a nested evolutionary relationship with strong supports (Fig. 8). Subclade *Hellenia* included *C. speciosus* Guangdong and *C. speciosus* var. *marginatus* with highly supported node values (Fig. 8). Therefore, based on the results of the phylogenetic relationships herein, these two species should be transferred to *Hellenia* with the names of *Hellenia speciosa* Guangdong and *Hellenia speciosa* var. *marginatus*, respectively. Additionally, *C. tonkinensis* Yunnan OP712650 was clustered in the subclade of *Parahellenia* (Fig. 8). This result was in agreement with a previous study [20], which supported the opinion that *Parahellenia* subclade should be recognized as a new genus. Consequently, *C. tonkinensis* Yunnan should be transferred to genus *Parahellenia* with the name of *Parahellenia tonkinensis* Yunnan. Finally, *C. viridis* was clustered with *C. barbatus* and *C. beckii* in the *Costus* clade, and it did not show close relationship with previously reported *H. viridis* OL688999 [20]. This might be because the two analyzed species were different from each other, but they used the same name *viridis*.

According to the divergence time estimation, the crown node age of Costaceae estimated here (Fig. 9) (30.5 Mya, 95% HPD: 14.9 − 49.3 Mya) was in close proximity to a previous study reported by Fu et al. [40] (24.9 Mya). However, Specht [49] using *trnL-F* and *trnK* sequence data of Costaceae, estimated the divergence time of Costaceae to be 65.6 ± 7.73 Mya; Kress et al. [50] using three gene regions (*rbcL*, *atpB*, and 18 S), estimated the crown diversification of Costaceae to be 52 ± 5 Mya; and André et al. [14] using nucleotide sequences of 2 plastid and 4 nuclear genetic markers, estimated the diversification of Costaceae around 50 Mya. These differences in age estimation of Costaceae may be caused by molecular data selection, taxon sampling, calibration point setting, and different methods of selection. In addition, our analyses also suggested that the main taxon within the *Costus* clade diverged at approximately 4.4 Mya (95% HPD: 1.5 − 10.8 Mya) (Fig. 9), which was in closeness to a previous report (4.6 Mya) [51].

## Conclusions

In this study, we analyzed and compared the structural characteristics of 13 complete chloroplast genomes of Costaceae, and estimated the phylogenetic divergence time of Costaceae. These 13 genomes had conserved quadripartite structure, similar protein-coding genes and codon usage, but also with some variations in genomes lengths, tRNA gene contents, introns, SSRs, long repeats and IR borders. Five highly divergent regions were identified, which would be useful for developing high-resolution DNA markers for further studies of Costaceae. Eight protein-coding genes (*cemA, clpP*, *ndhA*, *ndhF*, *petB*, *psbD*, *rps12* and *ycf1*) were found to undergo positive selection. Based on chloroplast genome sequences, the phylogenetic relationships in Costaceae showed that Costaceae species were divided into three clades, namely, a South American clade, an Asian clade and a *Costus* clade, with strongly supported values. Estimation of the divergence time of Costaceae suggested that the crown age of Costaceae was at approximately 30.5 Mya (95% HPD: 14.9 − 49.3 Mya). This study not only enriched the complete chloroplast genome resources of Costaceae, but also provided useful information for further studies of the evolution and phylogeny of Costaceae species.

## Methods

### Plant materials and DNA extraction

Due to sample collection challenges, samples of the eight Costaceae species, representing one *Monocostus* species from the South American clade (*M. uniflorus*), four *Costus* species (*C. barbatus*, *C. beckii*, *C. dubius*, and *C. woodsonii*) from the *Costus* clade, and three species (*C. speciosus* Guangdong, *C. speciosus* var. *marginatus*, and *C. tonkinensis* Yunnan) from the Asian clade (Fig. S2), were obtained from the resource garden of the environmental horticulture research institute (23°23′N, 113°26′E) at the Guangdong Academy of Agricultural Sciences, Guangzhou, China. Species formal identifications were made using the *Flora of China* [1], *The Zingiberaceous resources in China* [8], *Botanical paintings of Chinese Zingiberales* [52], and also conducted using photos (available on https://www.gingersrus.com/Costus.php). Young and healthy leaves of seedlings were collected and quickly frozen in liquid nitrogen and stored at -80 ℃ until use. The total genomic DNA was extracted from young leaves using sucrose gradient centrifugation method with minor modifications [53]. DNA integrity and quality were assessed by a NanoDrop 2000 microspectrometer (Wilmington, DE, USA), and detected using a 1% (w/v) agarose gel electrophoresis. The other five published complete chloroplast genomes of Costaceae were downloaded from NCBI for the following comparative analyses.

### Illumina sequencing, assembly and annotation

Each high-quality DNA sample was sheared into fragments of about 350 bp to construct a library according to the manufacturer’s instructions (New England Biolabs, Ipswich, MA, England). Sequencing was carried out on an Illumina NovaSeq 6000 platform with 150 bp paired-end reads length (Biozeron, Shanghai, China). The raw data were checked using FastQC v. 0.11.9 (http://www.bioinformatics.babraham.ac.uk/projects/fastqc/), and filtered by Trimmomatic v. 0.39 [54] with default parameters. Next, filtered reads were *de novo* assembled using GetOrganelle v. 1.7.6.1 [55] with default settings. Geneious Prime 2022 (Biomatters Ltd., Auckland, New Zealand) [56] was used to align the contigs and the start and stop codons were manually edited with a reference chloroplast genome of *C. viridis* (GenBank accession number MK262733). Then, each assembled chloroplast genome was annotated in GeSeq [57] and the online Dual Organellar Genome Annotator (DOGMA) [58] with default parameters, respectively. Additionally, tRNAscanSE v. 2.0.5 [59] and BLAST v. 2.13.0 [60] were used to confirm the tRNA and rRNA genes. The annotation results were also validated by comparing them with NCBI’s non-redundant (Nr) protein database, Gene Ontology (GO), Clusters of orthologous groups (COG) for eukaryotic complete genomes database, Kyoto Encyclopedia of Genes and Genomes (KEGG) Automatic Annotation Server (KAAS) (http://www.genome.jp/kegg/kaas/) [61] and SWISS-PROT databases. The physical maps of complete chloroplast genomes were drawn using Organellar Genome Draw (OGDRAW) v. 1.3.1 [62]. The eight newly annotated complete chloroplast genome sequences were first validated using online GB2sequin [63]. Then, the annotation results were further validated and formatted using Sequin v. 15.50 from NCBI, and submitted to GenBank (see Table 1 for accession numbers).

### Sequence analysis and statistics

Codon usage was analyzed by using MEGA v. 7.0 [64], and the relative synonymous codon usage (RSCU) and amino acid frequencies were calculated with default parameters. When the RSCU value is larger than 1, the codon is used more often than expected, while values less than 1 indicate its relative rarity [65, 66]. The clustered heat map of RSCU values of 13 complete Costaceae chloroplast genomes was conducted by R v. 4.0.2 [67].

The long repeats sequences, which included forward, palindrome, reverse and complement repeats, were detected using REPuter [68] with a minimal repeat size of 30 bp, a repeat identity of more than 90%, and a hamming distance of 3. In this study, due to the collection difficulties of original sequenced data for the five published chloroplast genomes of Costaceae, the possible effects by different assembled ways on detection SSRs were not considered. SSRs in the chloroplast genomes were detected via MISA-web [69] by setting the minimum number of repeats to 10, 5, 4, 3, 3 and 3 for mononucleotide, dinucleotide, trinucleotide, tetranucleotide, pentanucleotide and hexanucleotide, respectively.

### Genome comparison and sequence divergence analyses

The contraction and expansion of the IR regions were obtained by comparing the SC/IR borders and their adjacent genes of 13 complete Costaceae chloroplast genomes using IRscope [70]. The mVISTA program in the Shuffle-LAGAN mode [71] was employed to compare the complete chloroplast genomes divergence among 13 complete chloroplast genomes with the annotated chloroplast genome of *C. barbatus* as the reference. Additionally, the chloroplast genome of *C. barbatus* was compared to the other 12 whole chloroplast genomes of Costaceae using CGView Server [72]. GC distributions were measured based on GC skew using the equation: GC skew = (G-C)/(G + C). To analyze the sequence divergence of complete chloroplast genomes in Costaceae, the protein-coding and intergenic regions among these 13 complete chloroplast genomes were extracted and aligned using MAFFT v. 7.458 [73] with default parameters. Then, nucleotide variability (Pi) values were analyzed using DnaSP v. 6.12.03 [74]. The step size was set to 200 bp, and the window length was set to 600 bp. The protein-coding regions with Pi > 0.007, the intergenic regions with Pi > 0.025, the region length > 250 bp, and 4 universal chloroplast DNA markers including *trnL-exon1*-*trnL-exon2*, *trnK-exon1*-*rps16-exon2*, *matK*-*trnK-exon1* and *trnL-exon2*-*trnF*, were extracted and then analyzed individually to differentiate these Costaceae species (Additional file 7). The maximum likelihood (ML) tree was calculated by using the nucleotide substitution model of Tamura-Nei in MEGA v. 7.0 [64] with 1000 replicates. Additionally, variable and parsimony informative base sites of the LSC, SSC, IRa, IRb, and complete chloroplast genomes of these 13 genomes were also calculated using *C. barbatus* as the reference.

### Positive selection analysis

Selective pressure was analyzed for consensus 79 protein-coding genes among 13 complete chloroplast genomes of Costaceae. The nonsynonymous (dN) and synonymous (dS) substitution rates were calculated by using the CodeML program implemented in EasyCodeML [75]. First, each single protein-coding gene was extracted, their stop codons removed and aligned separately using ClustalW in MEGA v. 7.0 [64], followed by manual adjustment for abnormal alignments. Next, based on the alignments, the ML tree was constructed using MEGA v. 7.0 as an input tree. Six models were investigated to calculate the dN and dS ratios (ω) and the likelihood ratio tests (LRTs): M0 (one-ratio), M1a (nearly neutral), M2a (positive selection), M3 (discrete), M7 (β) and M8 (β & ω > 1). The positive selection models (M2a and M8) were used to detect positively selected sites based on both ω and LRTs values [76]. A bayes empirical bayes (BEB) method [77] was then selected to calculate posterior probabilities. In the BEB analysis, posterior probability higher than 0.95 and 0.99 indicated sites that were under positive selection and strong positive selection, respectively.

### Phylogenetic analysis

To reconstruct and confirm the phylogenetic relationships of *Hellenia* and *Parahellenia* in Costaceae, a total of 31 chloroplast genomes sequences of Costaceae were analyzed, which included 13 complete and 18 incomplete chloroplast genomes (Table S7). Of these 31 genomes, 8 complete chloroplast genomes were generated in the present study, and the other 23 chloroplast genomes sequences were obtained from the GenBank database and individuals (Table S7, Additional file 9), respectively. Twelve chloroplast genomes of the Zingiberaceae species in GenBank were added as outgroups (Table S7). The chloroplast genome sequences were aligned using the MAFFT v. 7.458 [73] with default parameters and manually checked when necessary. The best nucleotide substitution model (general-time-reversible, gamma distribution and invariable sites, GTR + G + I) was determined using the Akaike Information Criterion (AIC) in jModelTest v. 2.1.10 [78]. Subsequently, the ML tree was constructed using PhyML v. 3.0 [79], and a bootstrap test was performed with 1000 replicates to calculate the bootstrap values for all branch nodes. Bayesian inference (BI) analysis was carried out using MrBayes v. 3.2.6 [80]. Two Markov Chain Monte Carlo algorithm (MCMC) runs were performed with 200,000 generations and four Markov chains, starting from random trees, sampling trees every 100 generations, and discarding the first 10% of samples as burn-in. The phylogenetic trees were edited and visualized using iTOL v. 3.4.3 (http://itol.embl.de/itol.cgi).

#### Divergence time estimation

As some published chloroplast genomes of Costaceae missed large fragments, we only selected complete or nearly complete chloroplast genomes for divergence time estimation (Table S8). Divergence time estimation was performed by the dataset of 75 single-copy protein-coding genes shared in 22 chloroplast genomes of Costaceae using the MCMC tree in PAML v. 4.4 [81]. First, the best nucleotide substitution model (GTR) was selected using jModelTest v. 2.1.10 [78] under AIC, and construction ML tree from the chloroplast genomes sequences were undertaken using PhyML v. 3.0 [79]. Second, two fossil records and one calibration point was obtained and used in the divergence time estimation. *Zingiberopsis attenuate* [82] was used as a mean age of 65 Million years ago (Mya) for the crown age of family Zingiberaceae. *Ensete oregonense* [83] was applied to calibrate the crown age of *Ensete* and *Musella* with a mean age of 43 Mya. Each fossil calibration point was assumed to follow a normal distribution with a standard deviation of 2 and an offset of 2, resulting in 63.1 − 70.9, and 41.1 − 48.9 Mya 95% intervals, respectively. Then, one calibration point (http://www.timetree.org/) was also used in this analysis, including the calibration point between *Zingiber* and *Kaempferia* with a mean age of 6.86 Mya (3.0 − 10.0 Mya). Thirdly, the new ML tree constructed from chloroplast genomes sequences was used as a starting tree for the MCMC run. MCMC run was set at 400,000 generations, sampling every 100 generations, and removing the first 10% generations as burn in. Divergence time estimation was calculated by parameters of clock = 2 and model = 0, with 95% highest posterior density (HPD) intervals, and then inserting the resulting divergence times into the ML tree.

### Electronic supplementary material

Below is the link to the electronic supplementary material.

**Supplementary Material 1: Table S1.** The basic information of 8 newly sequenced chloroplast genomes in family Costaceae

**Supplementary Material 2: Table S2.** Genes distribution in 13 Costaceae complete chloroplast genomes

**Supplementary Material 3: Table S3.** Comparison of the long repeats among 13 Costaceae complete chloroplast genomes

**Supplementary Material 4: Table S4.** Statistics of simple sequence repeats (SSRs) sequences distribution and designed primers in 13 Costaceae complete chloroplast genomes

**Supplementary Material 5:** **Table S5**. Codon usages of all protein coding genes in 13 Costaceae complete chloroplast genomes

**Supplementary Material 6: Table S6.** Nucleotide diversity (Pi) analyses of protein coding genes, intron and intergenic regions in 13 Costaceae complete chloroplast genomes

**Supplementary Material 7:** Fasta form of 14 divergent regions and 4 universal chloroplast DNA markers sequences from 13 species/accessions of Costaceae

**Supplementary Material 8: Table S7.** Information of chloroplast genomes sequences used in present phylogenetic analyses

**Supplementary Material 9:** Fasta form of nine Costaceae nearly complete chloroplast genomes sequences provided by Dr. Juan Chen in South China Botanical Garden of Chinese Academy of Sciences

**Supplementary Material 10: Table S8.** Information of chloroplast genomes sequences used in divergence time estimation

**Supplementary Material 11: Fig. S1.** Maximum likelihood (ML) trees of 13 species/accessions of Costaceae based on the chloroplast genomes divergent genes and intergenic regions. a ML tree based on intergenic sequences of matK-trnK-exon1. b ML tree based on intergenic sequences of trnK-exon1-rps16-exon2. c ML tree based on intergenic sequences of trnL-exon1-trnL-exon2. d ML tree based on intergenic sequences of trnL-exon2-trnF. e ML tree based on sequences of gene ccsA. f ML tree based on sequences of gene ndhF. g ML tree based on sequences of gene rps3. h ML tree based on sequences of gene rps15. i ML tree based on sequences of gene ycf1-D2. j ML tree based on sequences of gene rpoC1-exon1. k ML tree based on the intergenic sequences of psaC-ndhE. l ML tree based on the intergenic sequences of ccsA-ndhD. m ML tree based on the intergenic sequences of rps15-ycf1-D2. n ML tree based on the intergenic sequences of atpH-atpI. o ML tree based on the intergenic sequences of accD-psaI. p ML tree based on the intergenic sequences of trnS-trnG-exon1. q ML tree based on the intergenic sequences of rpl32-trnL. r ML tree based on the intergenic sequences of rpl16-exon2-rpl16-exon1. s ML tree based on the intergenic sequences of ccsA-ndhD+rpl16-exon2-rpl16-exon1. t ML tree based on the intergenic sequences of ccsA-ndhD+ rps15-ycf1-D2. u ML tree based on the intergenic sequences of rps15-ycf1-D2+ rpl16-exon2-rpl16-exon1. v ML tree based on the intergenic sequences of ccsA-ndhD+rps15-ycf1-D2+ rpl16-exon2-rpl16-exon1. w ML tree based on the intergenic sequences of ycf1-D2+ ndhF

**Supplementary Material 12: Fig. S2.** Comparison of morphologies among eight species of family Costaceae. (A) terminally flowering of Costus barbatus, (B) terminally flowering of Costus speciosus Guangdong, (C) leaf morphology of Costus tonkinensis Yunnan, (D) basally flowering of Costus dubius, (E) leaf morphology of Costus speciosus var. marginatus, (F) terminally flowering of Costus woodsonii, (G) basally flowering of Costus beckii, (H) terminally flowering of C. beckii, and (I) flowering of Monocostus uniflorus

## Data Availability

All raw read data are available at the Sequence Read Archive (SRA) with the BioProject accession number PRJNA882627 (https://www.ncbi.nlm.nih.gov/bioproject/882627). The eight newly sequenced complete chloroplast genomes in this study have been submitted to GenBank (https://www.ncbi.nlm.nih.gov) with accession numbers OP712648 - OP712655 and available in NCBI (https://www.ncbi.nlm.nih.gov/) (see Table 1). All voucher specimens were deposited in the resource garden of the Environmental Horticulture Research Institute, Guangdong Academy of Agricultural Sciences, Guangzhou, China. The nine nearly complete chloroplast genome sequences of Costaceae provided by Dr. Juan Chen and their accession numbers are listed in Additional file 9. Other chloroplast genome sequences for phylogenetic analyses and divergence time estimation can be obtained from NCBI, and their accession numbers are listed in Table S7 and Table S8.
