# Supplementary material for: Thirteen complete chloroplast genomes of the costaceae family: insights into genome structure, selective pressure and phylogenetic relationships
Source: BMC Genomics. 2024 Jan 17;25:68. doi: 10.1186/s12864-024-09996-4 (PMC10792896; doi:10.1186/s12864-024-09996-4)
Supplement: Supplementary file 6 — Supplementary Material 6: Table S6. Nucleotide diversity (Pi) analyses of protein coding genes, intron and intergenic regions in 13 Costaceae complete chloroplast genomes [file 12864_2024_9996_MOESM6_ESM.docx]

**Table S7.** Information of chloroplast genomes sequences used in present phylogenetic analyses.

| Sample number/Sequence source | Species name | Genbank accession number | Note |
| --- | --- | --- | --- |
| CO-1/the present study assembled | *Costus barbatus* | OP712648 | complete genome |
| CO-8/the present study assembled | *Costus beckii* | OP712653 | complete genome |
| CO-6/the present study assembled | *Costus dubius* | OP712651 | complete genome |
| CO-2/the present study assembled | *Costus speciosus* Guangdong | OP712649 | complete genome |
| CO-7/the present study assembled | *Costus speciosus* var. *marginatus* | OP712652 | complete genome |
| CO-5/the present study assembled | *Costus tonkinensis* Yunnan | OP712650 | complete genome |
| Genbank | *Costus viridis* | MK262733 | complete genome |
| CO-10/the present study assembled | *Costus woodsonii* | OP712654 | complete genome |
| Provided by Dr. Juan Chen | *Hellenia deliniana* YNPB | OL689000 | incomplete genome |
| Provided by Dr. Juan Chen | *Hellenia oblonga* YNYJ | OL688997 | incomplete genome |
| Genbank | *Hellenia speciosa* Guizhou | OK641589 | complete genome |
| Provided by Dr. Juan Chen | *Hellenia speciosa* GDGZ | OL688995 | incomplete genome |
| Provided by Dr. Juan Chen | *Hellenia viridis* YNRL | OL688999 | incomplete genome |
| CO-11/the present study assembled | *Monocostus uniflorus* | OP712655 | complete genome |
| Provided by Dr. Juan Chen | *Parahellenia tonkinensis* GXJX | OL688994 | incomplete genome |
| Provided by Dr. Juan Chen | *Parahellenia tonkinensis* VN | OL688993 | incomplete genome |
| Provided by Dr. Juan Chen | *Parahellenia tonkinensis* YNMLP | OL688992 | incomplete genome |
| Provided by Dr. Juan Chen | *Parahellenia malipoensis* | OL688996 | incomplete genome |
| Provided by Dr. Juan Chen | *Parahellenia yunnanensis* | OL688998 | incomplete genome |
| Genbank | *Costus gabonensis* | MH603407 | incomplete genome |
| Genbank | *Costus osae* | MH603408 | incomplete genome |
| Genbank | *Costus pictus* | MH603409 | incomplete genome |
| Genbank | *Costus pulverulentus* | KF601573 | incomplete genome |
| Genbank | *Chamaecostus acaulis* | MH603404 | incomplete genome |
| Genbank | *Dimerocostus strobilaceus* | MH603413 | incomplete genome |
| Genbank | *Tapeinochilos ananassae* | MH603446 | incomplete genome |
| Genbank | *Hellenia lacera* | ON598391 | complete genome |
| Genbank | *Hellenia speciosa* Yunnan | ON598392 | complete genome |
| Genbank | *Costus tonkinensis* | ON598393 | complete genome |
| Genbank | *Costus gabonensis* | MH603407 | incomplete genome |
| Genbank | *Costus osae* | MH603408 | incomplete genome |
| Genbank | *Costus pictus* | MH603409 | incomplete genome |
| Genbank | *Costus dubius* | MH603406 | incomplete genome |
| Genbank | *Monocostus uniflorus* | KF601572 | incomplete genome |
| Genbank | *Zingiber officinale* | NC_044775 | complete genome |
| Genbank | *Zingiber montanum* | MK262727 | complete genome |
| Genbank | *Kaempferia elegans* | MK209002 | complete genome |
| Genbank | *Kaempferia galanga* | MK209001 | complete genome |
| Genbank | *Hedychium coronarium* Guangdong | MK262736 | complete genome |
| Genbank | *Hedychium neocarneum* | MT473709 | complete genome |
| Genbank | *Globba marantina* | MT473705 | complete genome |
| Genbank | *Globba multiflora* | MT473706 | complete genome |

**Table S7.** continued.

| Sample number/Sequence source | Species name | Genbank accession number | Note |
| --- | --- | --- | --- |
| Genbank | *Globba schomburgkii* | MK262735 | complete genome |
| Genbank | *Cautleya gracilis* | MW769781 | complete genome |
| Genbank | *Roscoea humeana* | NC_046582 | complete genome |
| Genbank | *Roscoea tibetica* | NC_047420 | complete genome |
