# Supplementary material for: Thirteen complete chloroplast genomes of the costaceae family: insights into genome structure, selective pressure and phylogenetic relationships
Source: BMC Genomics. 2024 Jan 17;25:68. doi: 10.1186/s12864-024-09996-4 (PMC10792896; doi:10.1186/s12864-024-09996-4)
Supplement: Supplementary file 9 — Supplementary Material 9: Fasta form of nine Costaceae nearly complete chloroplast genomes sequences provided by Dr. Juan Chen in South China Botanical Garden of Chinese Academy of Sciences [file 12864_2024_9996_MOESM9_ESM.docx]

**Table S8.** Information of chloroplast genomes sequences used in divergence time estimation.

| Sample number/Sequence source | Species name | Genbank accession number | Note |
| --- | --- | --- | --- |
| CO-1/the present study assembled | *Costus barbatus* | OP712648 | complete genome |
| CO-8/the present study assembled | *Costus beckii* | OP712653 | complete genome |
| CO-6/the present study assembled | *Costus dubius* | OP712651 | complete genome |
| CO-2/the present study assembled | *Costus speciosus* Guangdong | OP712649 | complete genome |
| CO-7/the present study assembled | *Costus speciosus* var. *marginatus* | OP712652 | complete genome |
| CO-5/the present study assembled | *Costus tonkinensis* Yunnan | OP712650 | complete genome |
| Genbank | *Costus viridis* | MK262733 | complete genome |
| CO-10/the present study assembled | *Costus woodsonii* | OP712654 | complete genome |
| Provided by Dr. Juan Chen | *Hellenia deliniana* YNPB | OL689000 | incomplete genome |
| Provided by Dr. Juan Chen | *Hellenia oblonga* YNYJ | OL688997 | incomplete genome |
| Genbank | *Hellenia speciosa* Guizhou | OK641589 | complete genome |
| Provided by Dr. Juan Chen | *Hellenia speciosa* GDGZ | OL688995 | incomplete genome |
| Provided by Dr. Juan Chen | *Hellenia viridis* YNRL | OL688999 | incomplete genome |
| CO-11/the present study assembled | *Monocostus uniflorus* | OP712655 | complete genome |
| Provided by Dr. Juan Chen | *Parahellenia tonkinensis* GXJX | OL688994 | incomplete genome |
| Provided by Dr. Juan Chen | *Parahellenia tonkinensis* VN | OL688993 | incomplete genome |
| Provided by Dr. Juan Chen | *Parahellenia tonkinensis* YNMLP | OL688992 | incomplete genome |
| Provided by Dr. Juan Chen | *Parahellenia malipoensis* | OL688996 | incomplete genome |
| Provided by Dr. Juan Chen | *Parahellenia yunnanensis* | OL688998 | incomplete genome |
| Genbank | *Hellenia lacera* | ON598391 | complete genome |
| Genbank | *Hellenia speciosa* Yunnan | ON598392 | complete genome |
| Genbank | *Costus tonkinensis* | ON598393 | complete genome |
| Genbank | *Zingiber officinale* | NC_044775 | complete genome |
| Genbank | *Zingiber montanum* | MK262727 | complete genome |
| Genbank | *Kaempferia elegans* | MK209002 | complete genome |
| Genbank | *Kaempferia galanga* | MK209001 | complete genome |
| Genbank | *Hedychium coronarium* Guangdong | MK262736 | complete genome |
| Genbank | *Hedychium neocarneum* | MT473709 | complete genome |
| Genbank | *Globba marantina* | MT473705 | complete genome |
| Genbank | *Globba multiflora* | MT473706 | complete genome |
| Genbank | *Globba schomburgkii* | MK262735 | complete genome |
| Genbank | *Cautleya gracilis* | MW769781 | complete genome |
| Genbank | *Roscoea humeana* | NC_046582 | complete genome |
| Genbank | *Roscoea tibetica* | NC_047420 | complete genome |
| Genbank | *Musella lasiocarpa* | NC_035637 | complete genome |
| Genbank | *Ensete glaucum* | LC610748 | complete genome |
| Genbank | *Ensete livingstonianum* | NC_058943 | complete genome |
| Genbank | *Ensete ventricosum* | OK012328 | complete genome |
