# Supplementary material for: Thirteen complete chloroplast genomes of the costaceae family: insights into genome structure, selective pressure and phylogenetic relationships
Source: BMC Genomics. 2024 Jan 17;25:68. doi: 10.1186/s12864-024-09996-4 (PMC10792896; doi:10.1186/s12864-024-09996-4)
Supplement: Supplementary file 11 — Supplementary Material 11: Fig. S1. Maximum likelihood (ML) trees of 13 species/accessions of Costaceae based on the chloroplast genomes divergent genes and intergenic regions. a ML tree based on intergenic sequences of matK-trnK-exon1. b ML tree based on intergenic sequences of trnK-exon1-rps16-exon2. c ML tree based on intergenic sequences of trnL-exon1-trnL-exon2. d ML tree based on intergenic sequences of trnL-exon2-trnF. e ML tree based on sequences of gene ccsA. f ML tree based on sequences of gene ndhF. g ML tree based on sequences of gene rps3. h ML tree based on sequences of gene rps15. i ML tree based on sequences of gene ycf1-D2. j ML tree based on sequences of gene rpoC1-exon1. k ML tree based on the intergenic sequences of psaC-ndhE. l ML tree based on the intergenic sequences of ccsA-ndhD. m ML tree based on the intergenic sequences of rps15-ycf1-D2. n ML tree based on the intergenic sequences of atpH-atpI. o ML tree based on the intergenic sequences of accD-psaI. p ML tree based on the intergenic sequences of trnS-trnG-exon1. q ML tree based on the intergenic sequences of rpl32-trnL. r ML tree based on the intergenic sequences of rpl16-exon2-rpl16-exon1. s ML tree based on the intergenic sequences of ccsA-ndhD+rpl16-exon2-rpl16-exon1. t ML tree based on the intergenic sequences of ccsA-ndhD+ rps15-ycf1-D2. u ML tree based on the intergenic sequences of rps15-ycf1-D2+ rpl16-exon2-rpl16-exon1. v ML tree based on the intergenic sequences of ccsA-ndhD+rps15-ycf1-D2+ rpl16-exon2-rpl16-exon1. w ML tree based on the intergenic sequences of ycf1-D2+ ndhF [file 12864_2024_9996_MOESM11_ESM.docx]

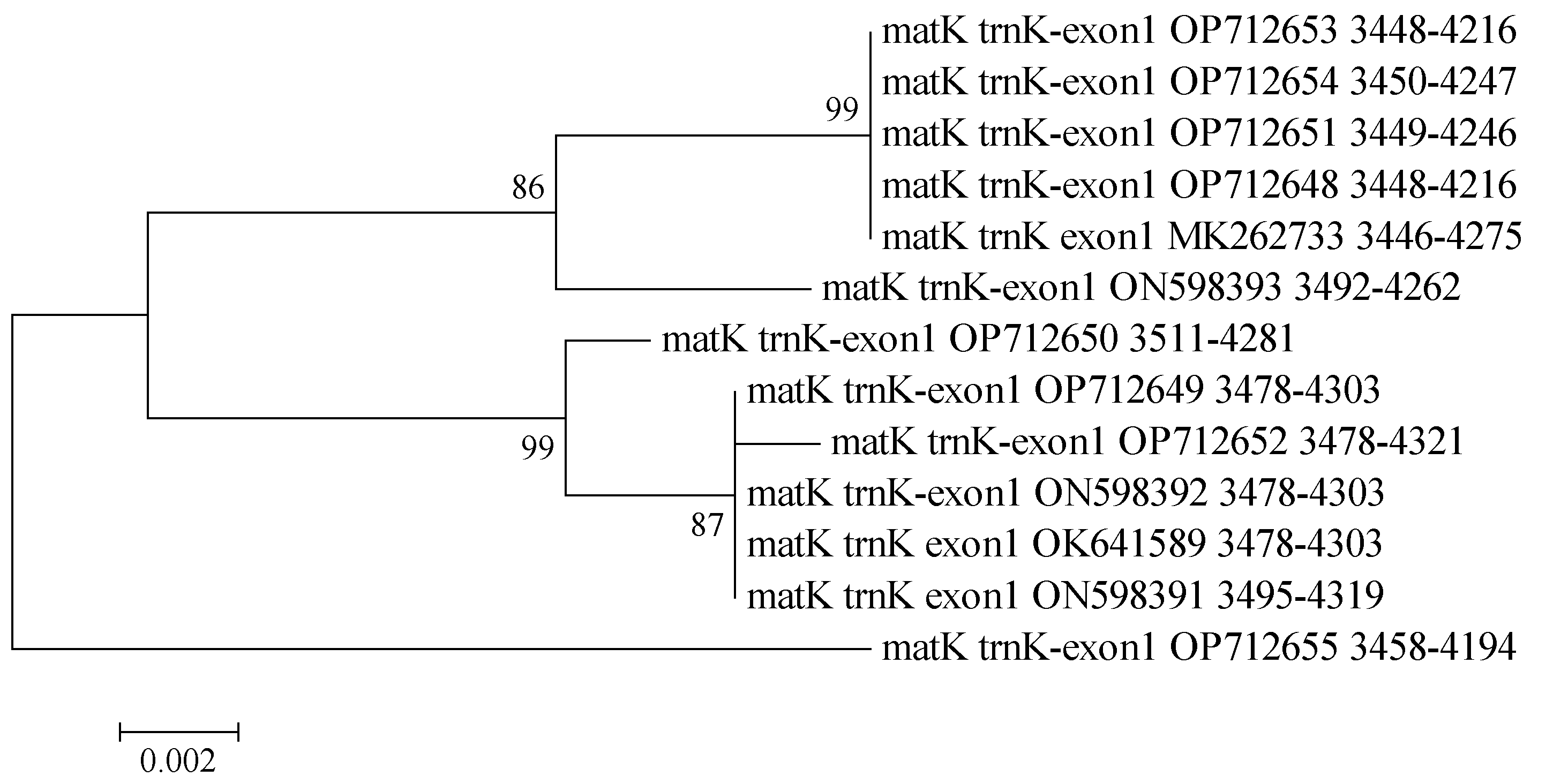


**a**

**
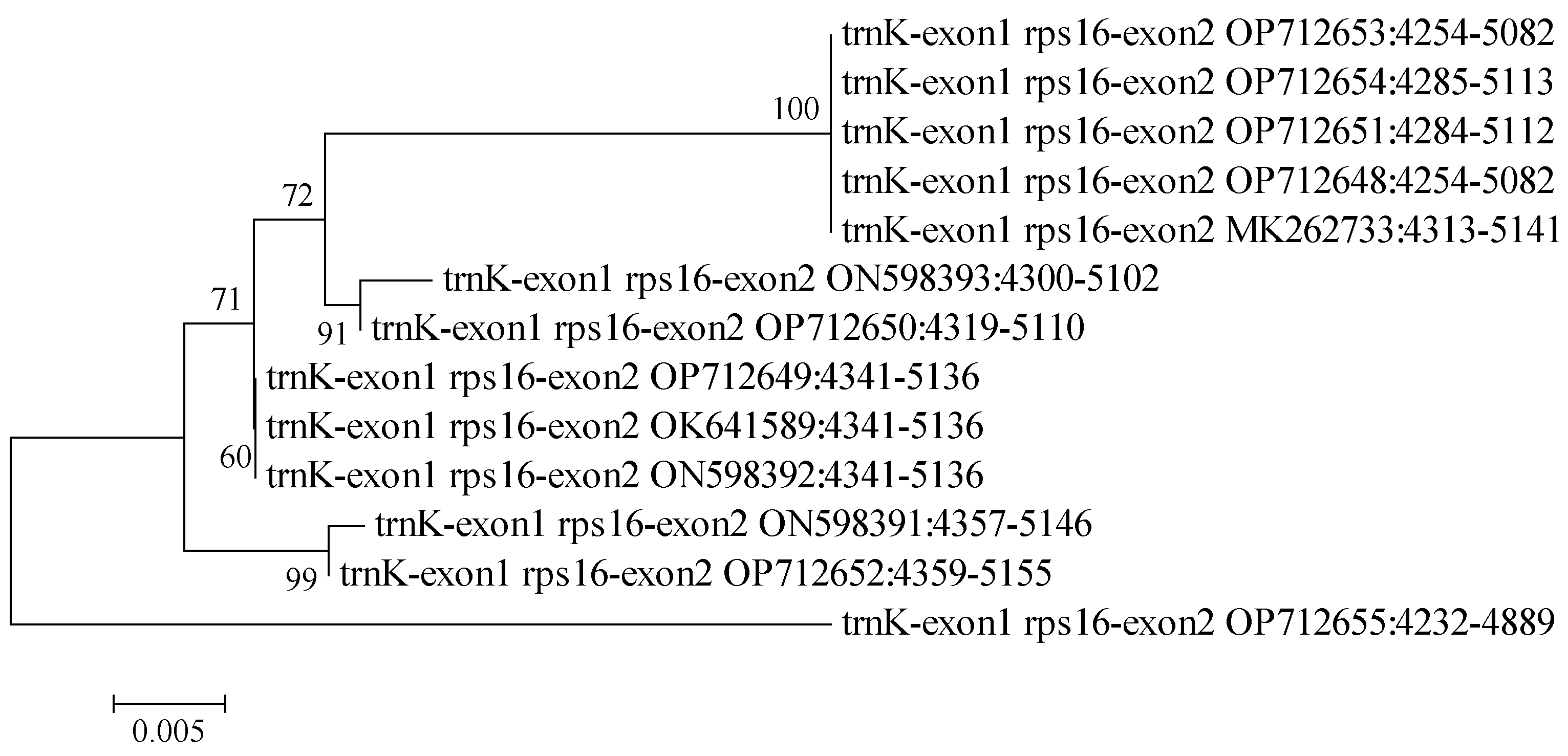
**

**b**

**Fig. S1.** Maximum likelihood (ML) trees of 13 species/accessions of Costaceae based on the chloroplast genomes divergent genes and intergenic regions. **a** ML tree based on intergenic sequences of *matK-trnK-exon1*. **b** ML tree based on intergenic sequences of *trnK-exon1-rps16-exon2*. **c** ML tree based on intergenic sequences of *trnL-exon1-trnL-exon2*. **d** ML tree based on intergenic sequences of *trnL-exon2-trnF*. **e** ML tree based on sequences of gene *ccsA*. **f** ML tree based on sequences of gene *ndhF*. **g** ML tree based on sequences of gene *rps3*. **h** ML tree based on sequences of gene *rps15*. **i** ML tree based on sequences of gene *ycf1-D2*. **j** ML tree based on sequences of gene *rpoC1-exon1*. **k** ML tree based on the intergenic sequences of *psaC-ndhE*. **l** ML tree based on the intergenic sequences of *ccsA-ndhD*. **m** ML tree based on the intergenic sequences of *rps15-ycf1-D2.* **n** ML tree based on the intergenic sequences of *atpH-atpI*. **o** ML tree based on the intergenic sequences of *accD-psaI*. **p** ML tree based on the intergenic sequences of *trnS-trnG-exon1*. **q** ML tree based on the intergenic sequences of *rpl32-trnL*. **r** ML tree based on the intergenic sequences of *rpl16-exon2-rpl16-exon1*. **s** ML tree based on the intergenic sequences of *ccsA-ndhD*+*rpl16-exon2-rpl16-exon1*. **t** ML tree based on the intergenic sequences of *ccsA-ndhD*+ *rps15-ycf1-D2*. **u** ML tree based on the intergenic sequences of *rps15-ycf1-D2*+*rpl16-exon2-rpl16-exon1*. **v** ML tree based on the intergenic sequences of *ccsA-ndhD*+*rps15-ycf1-D2*+ *rpl16-exon2-rpl16-exon1*. **w** ML tree based on the intergenic sequences of *ycf1-D2*+ *ndhF*.


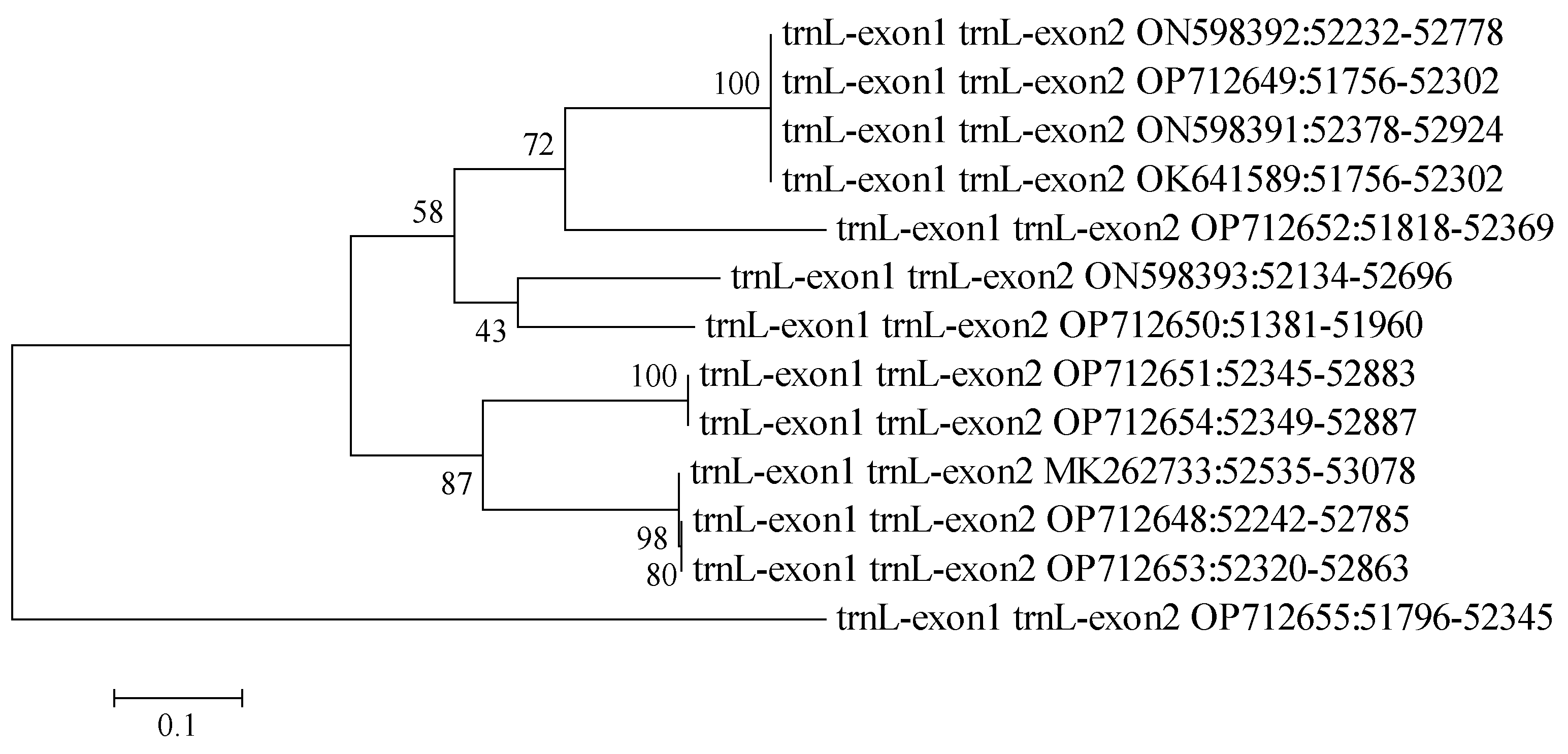


**c**


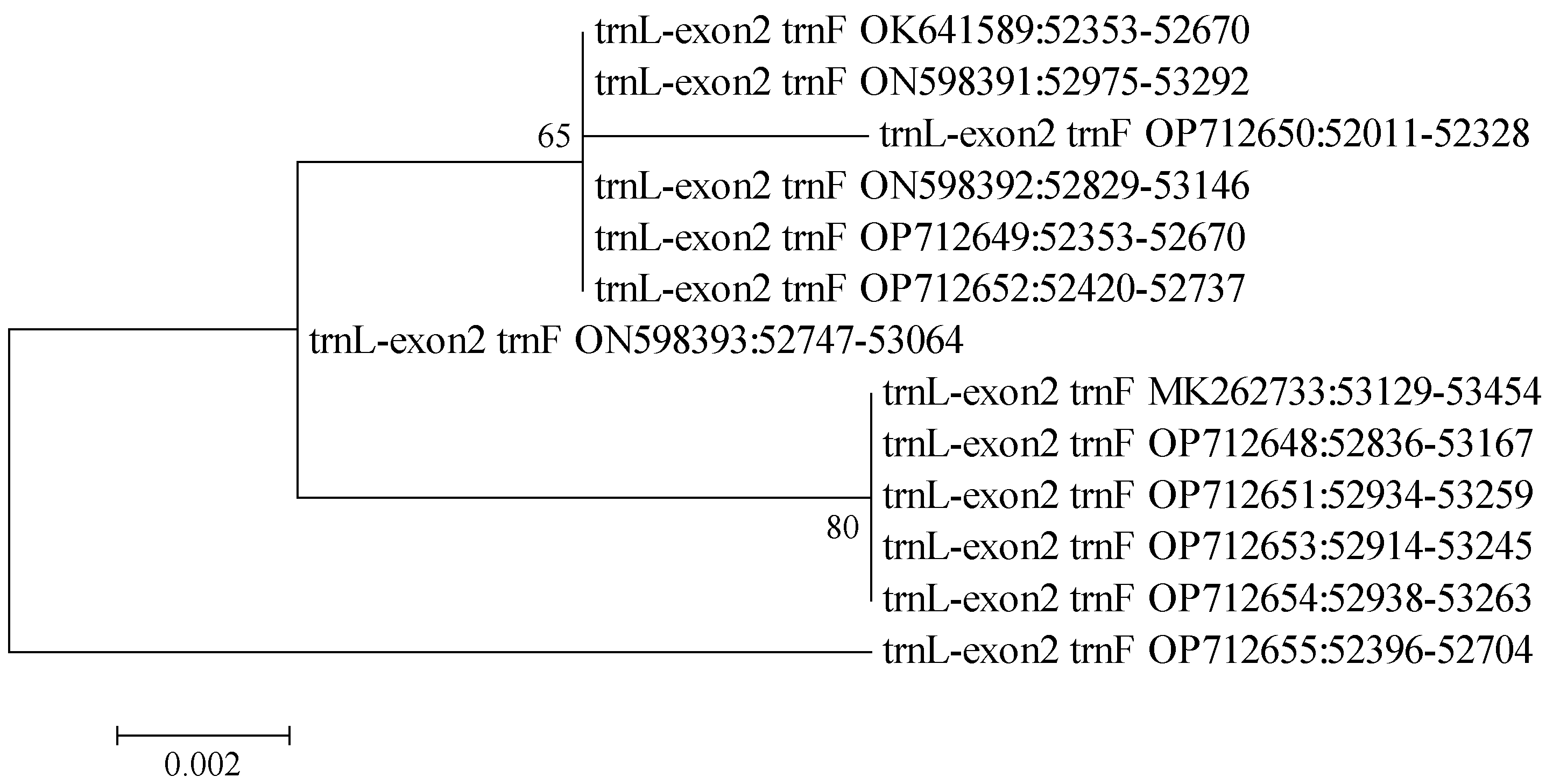


**d**

**Fig. S1** Continued.


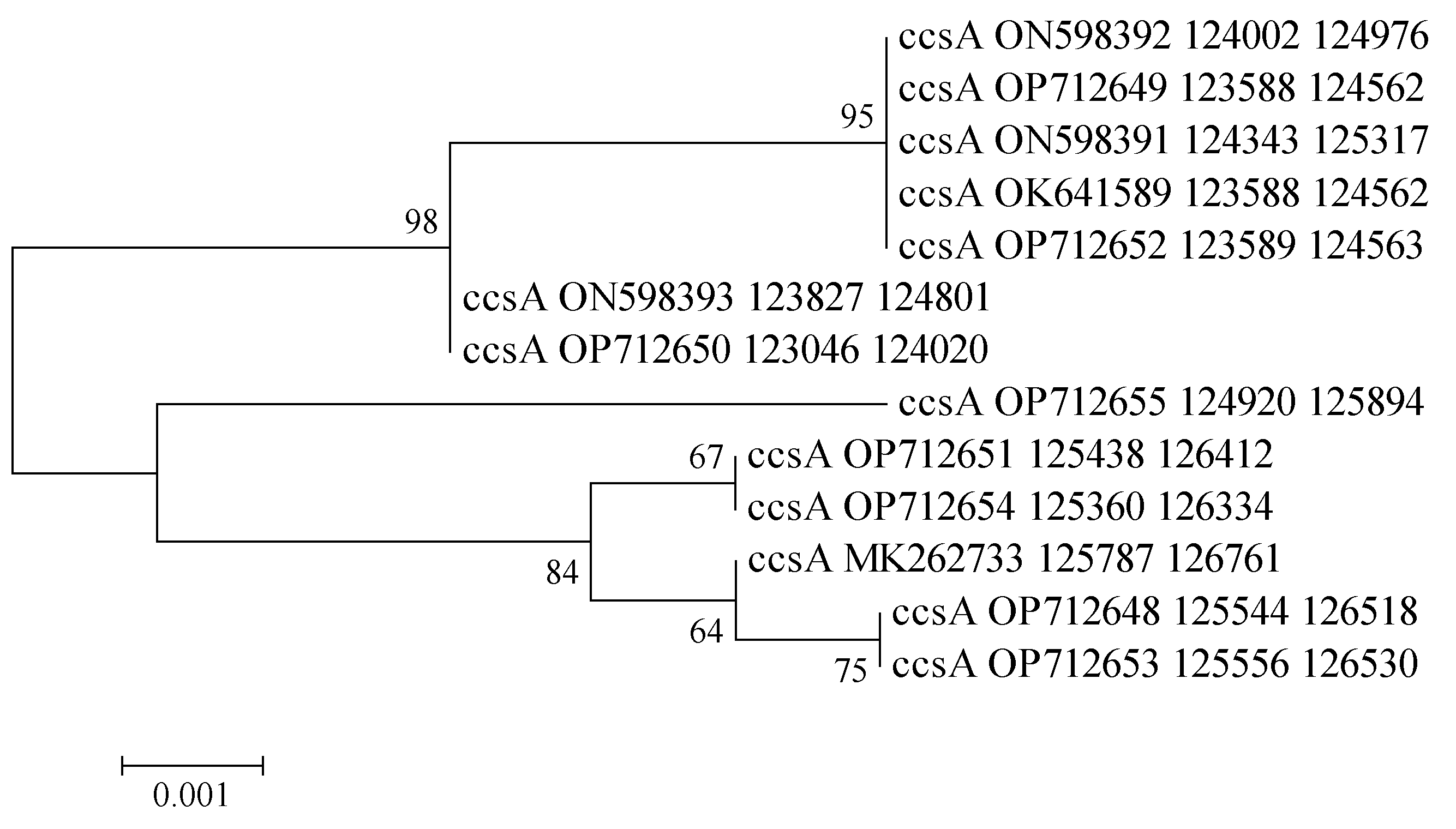


**e**


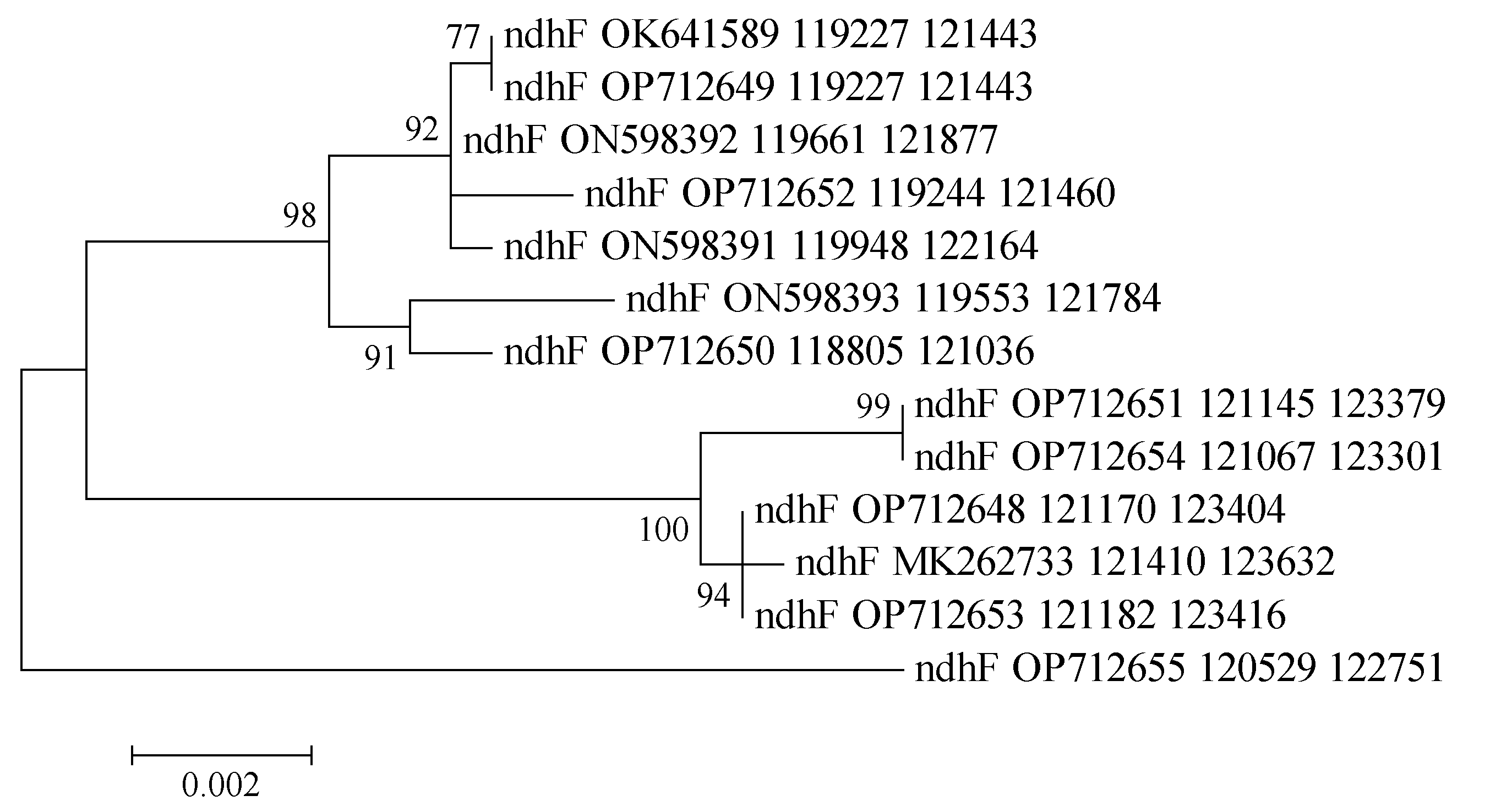


**f**


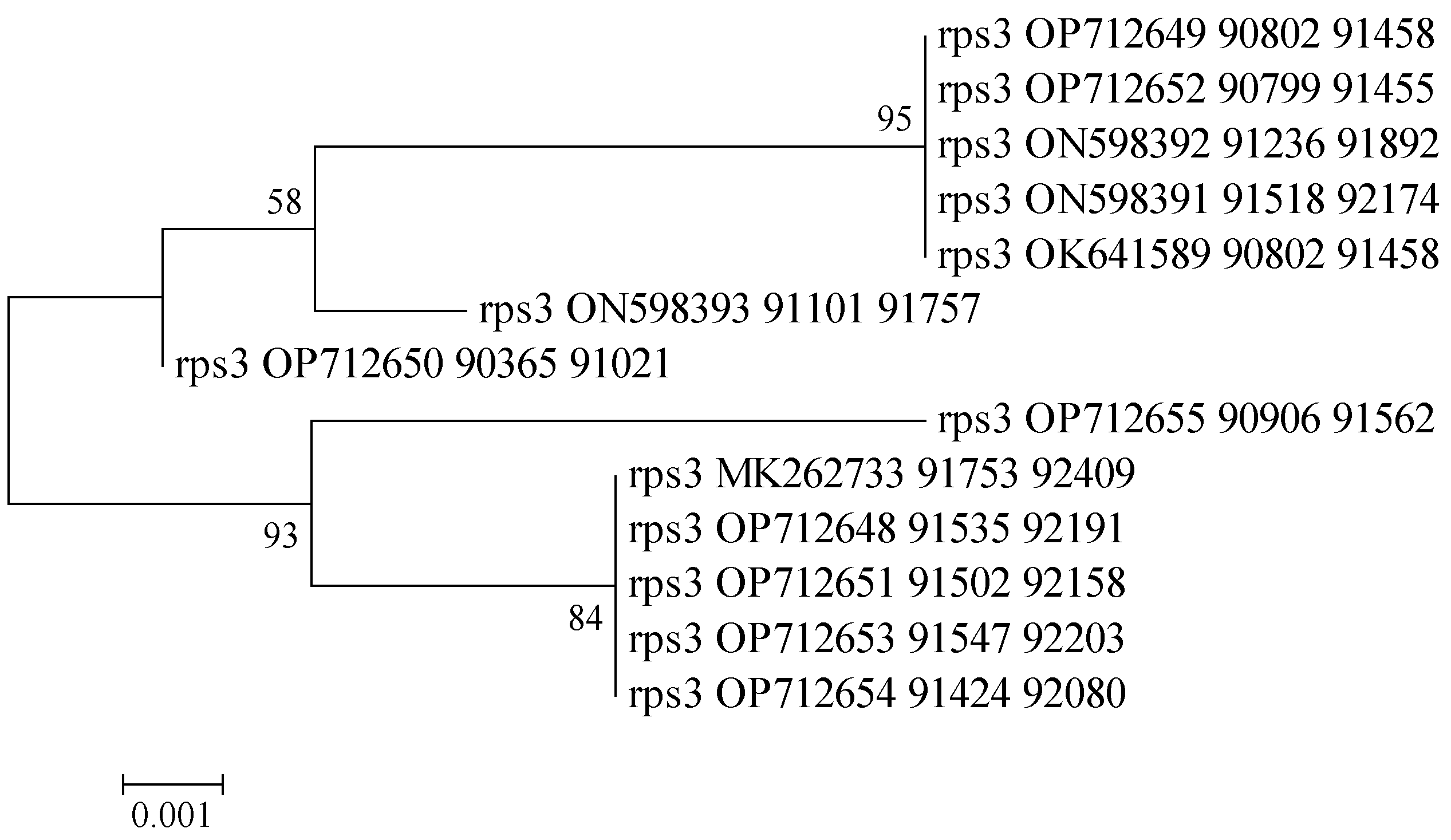


**g**

**Fig. S1** Continued.


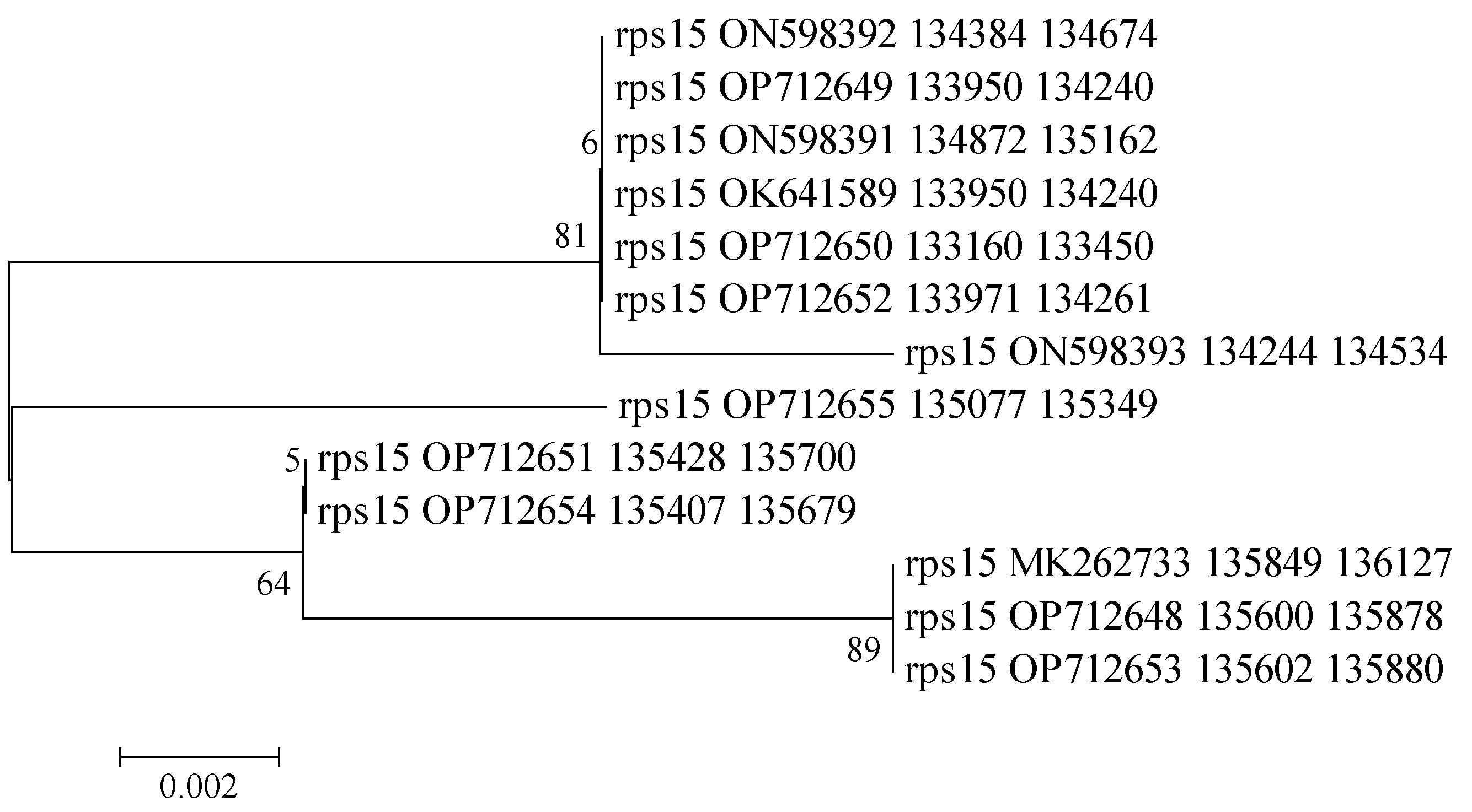

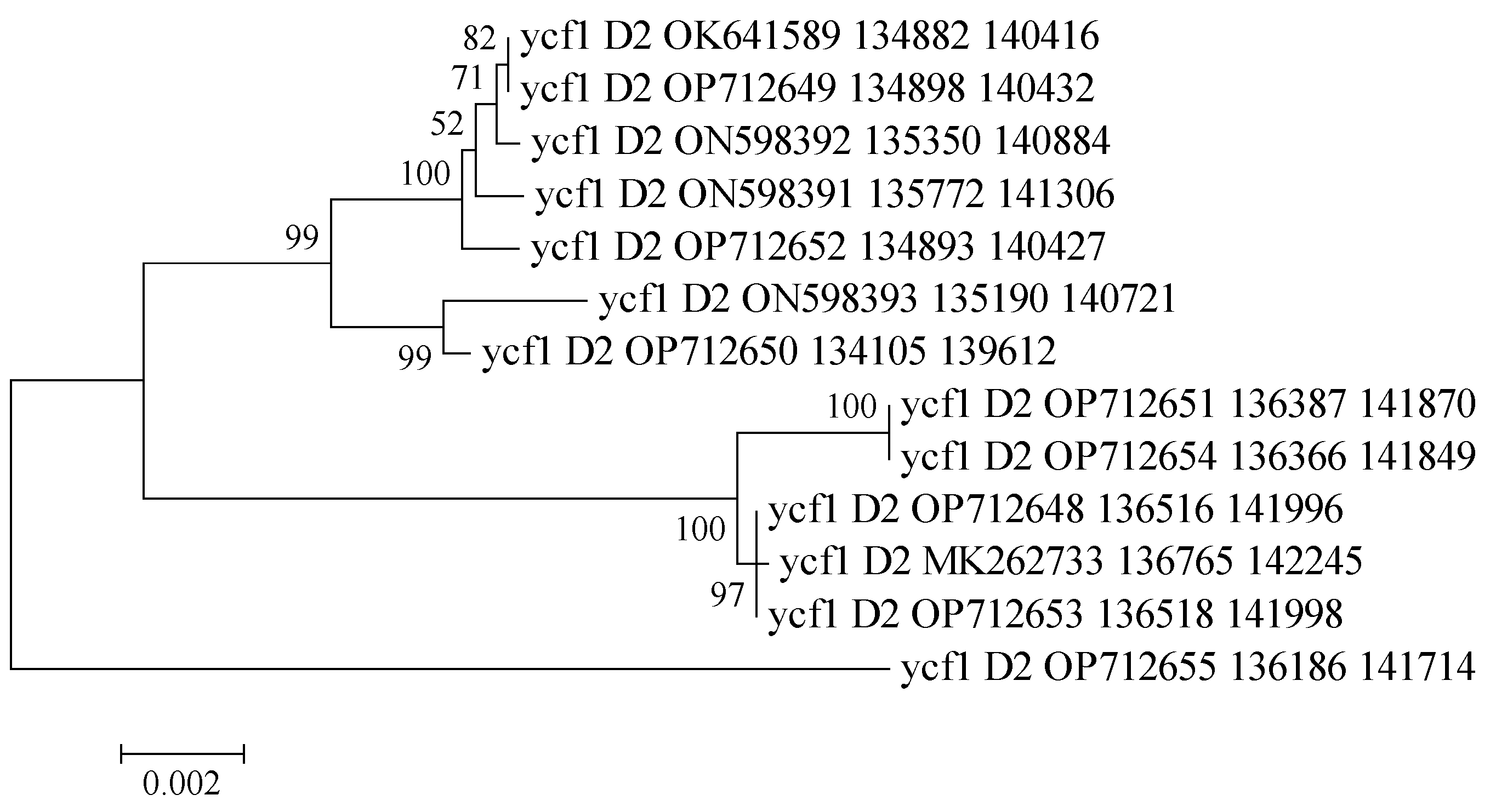


**i**

**h**


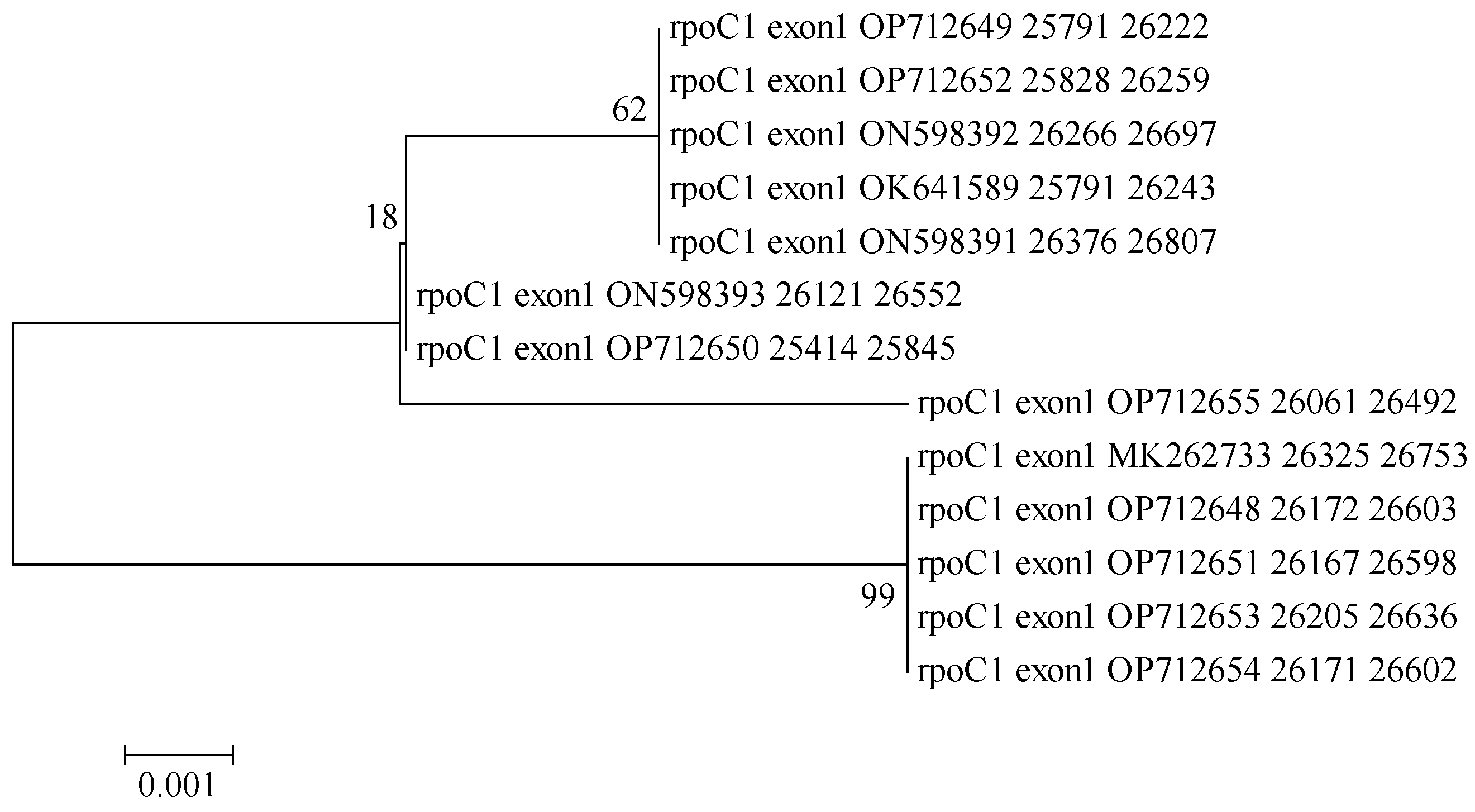


**j**

**Fig. S1** Continued.


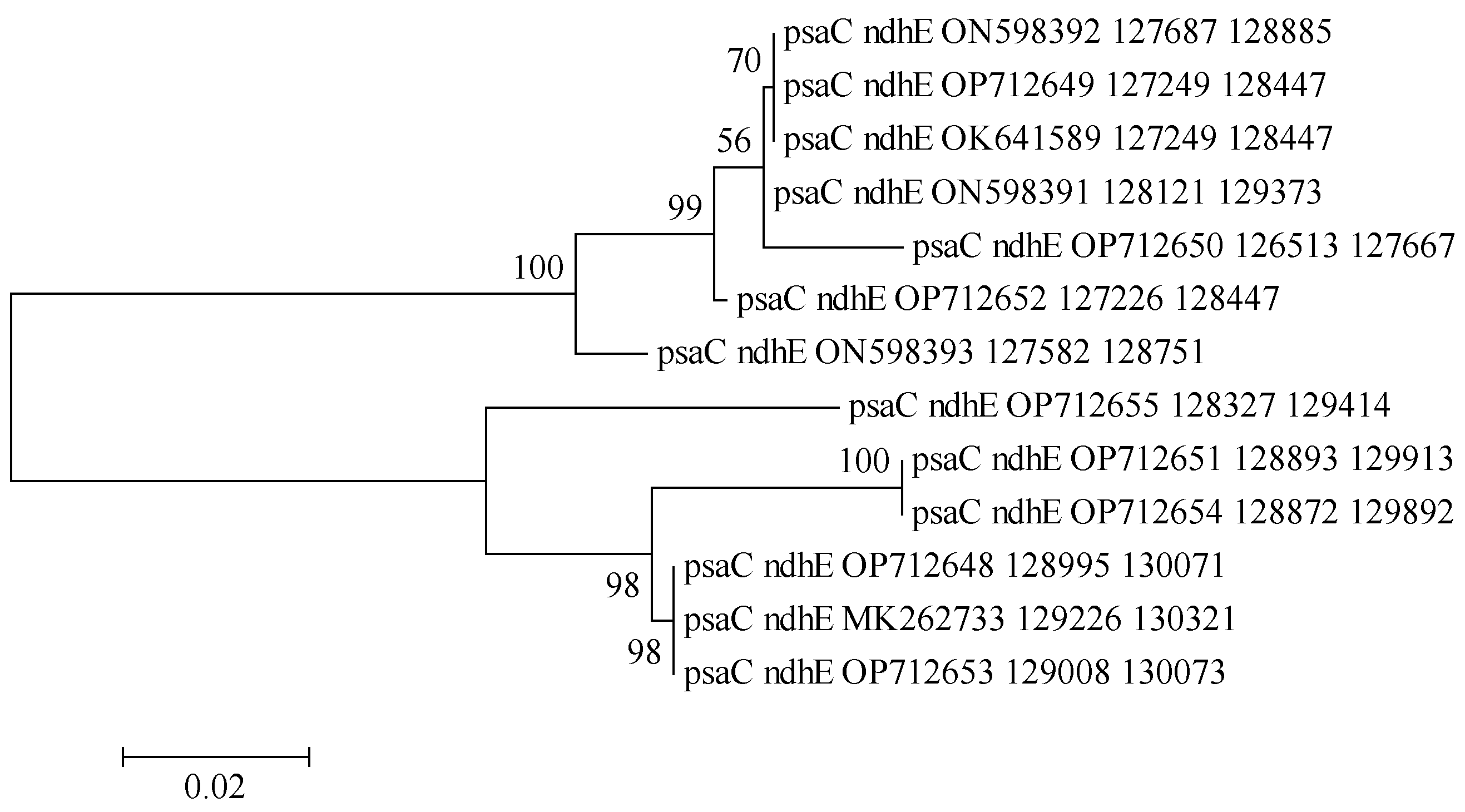


**k**


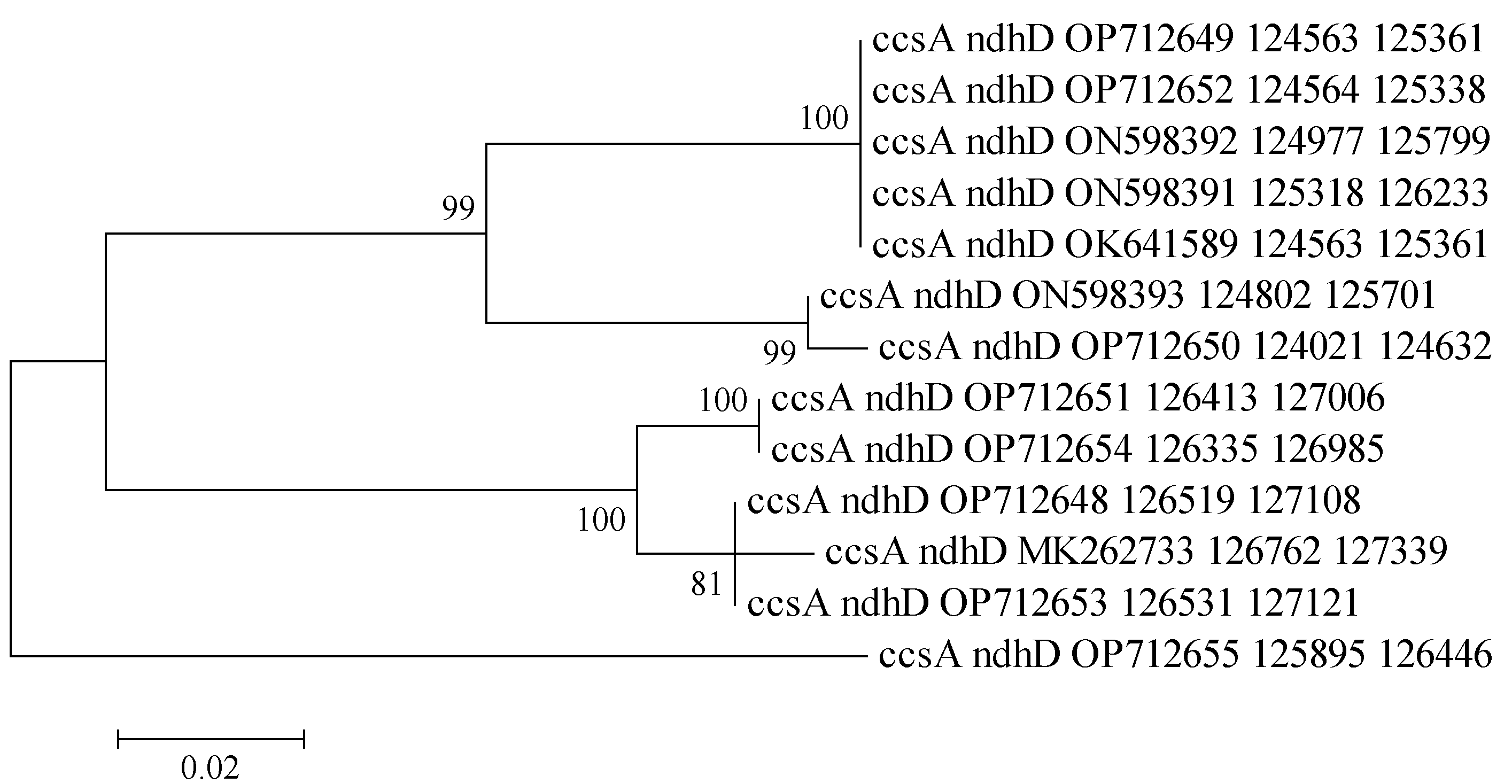


**l**


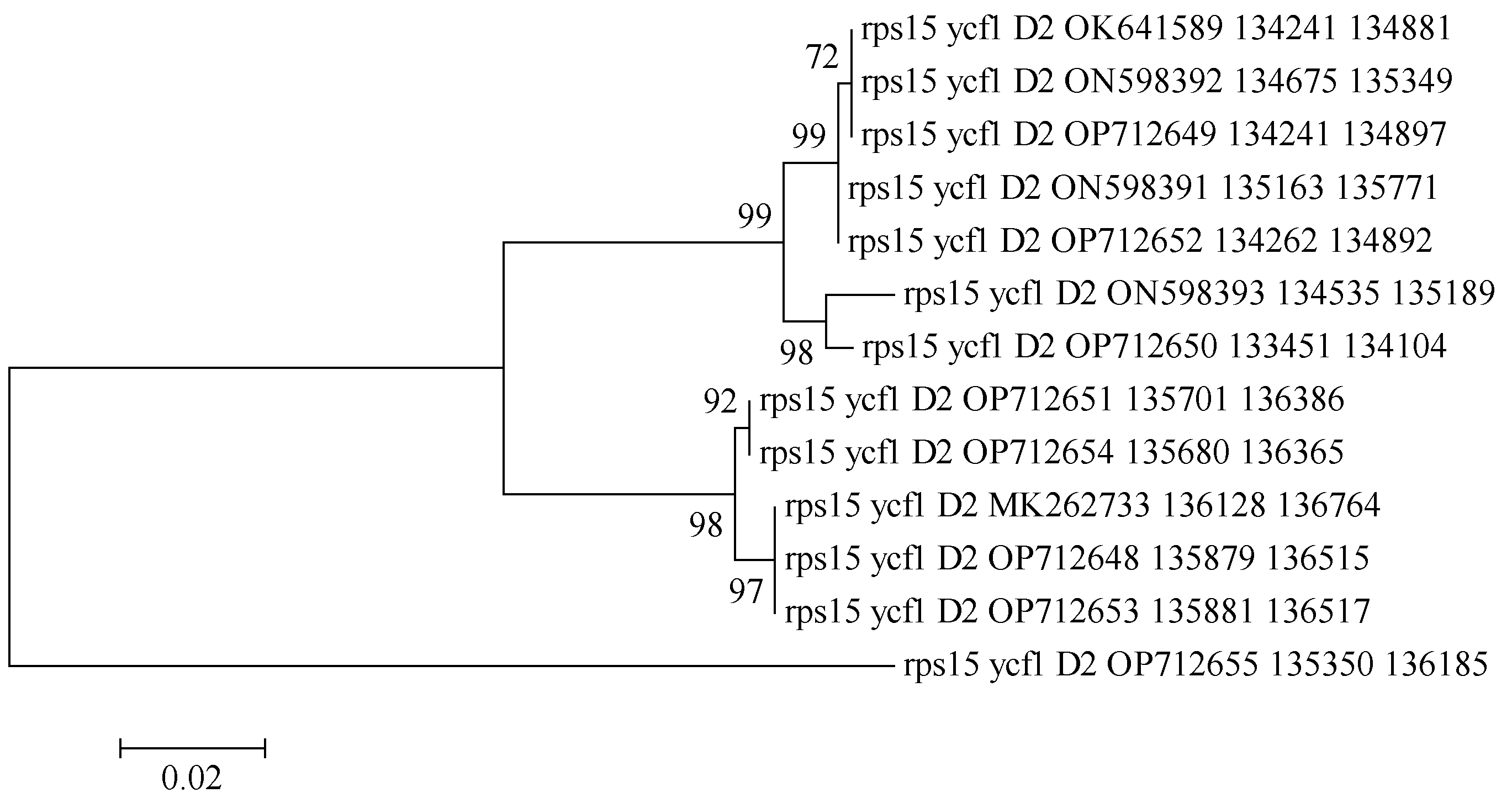


**m**

**Fig. S1** Continued.


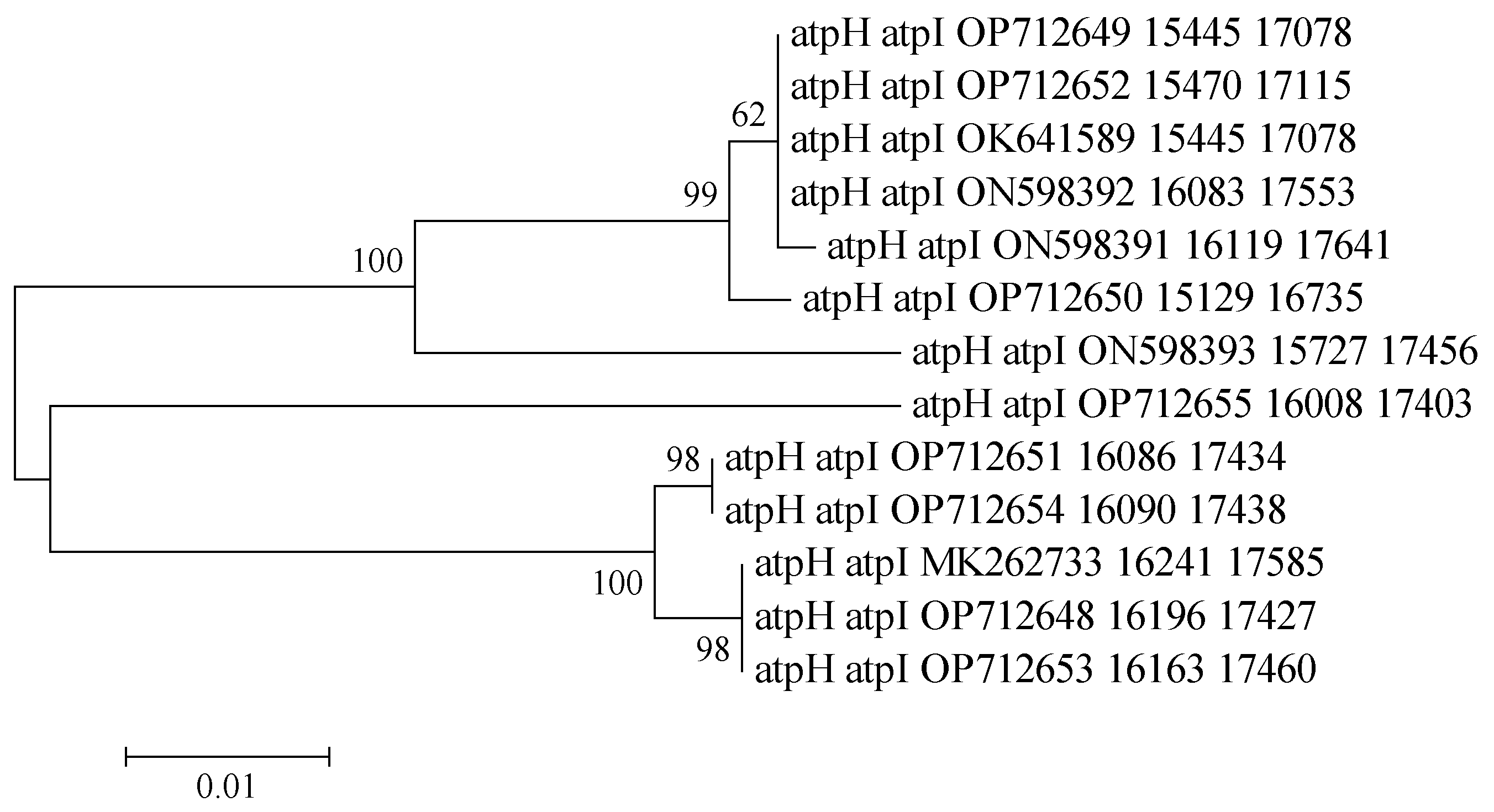


**n**


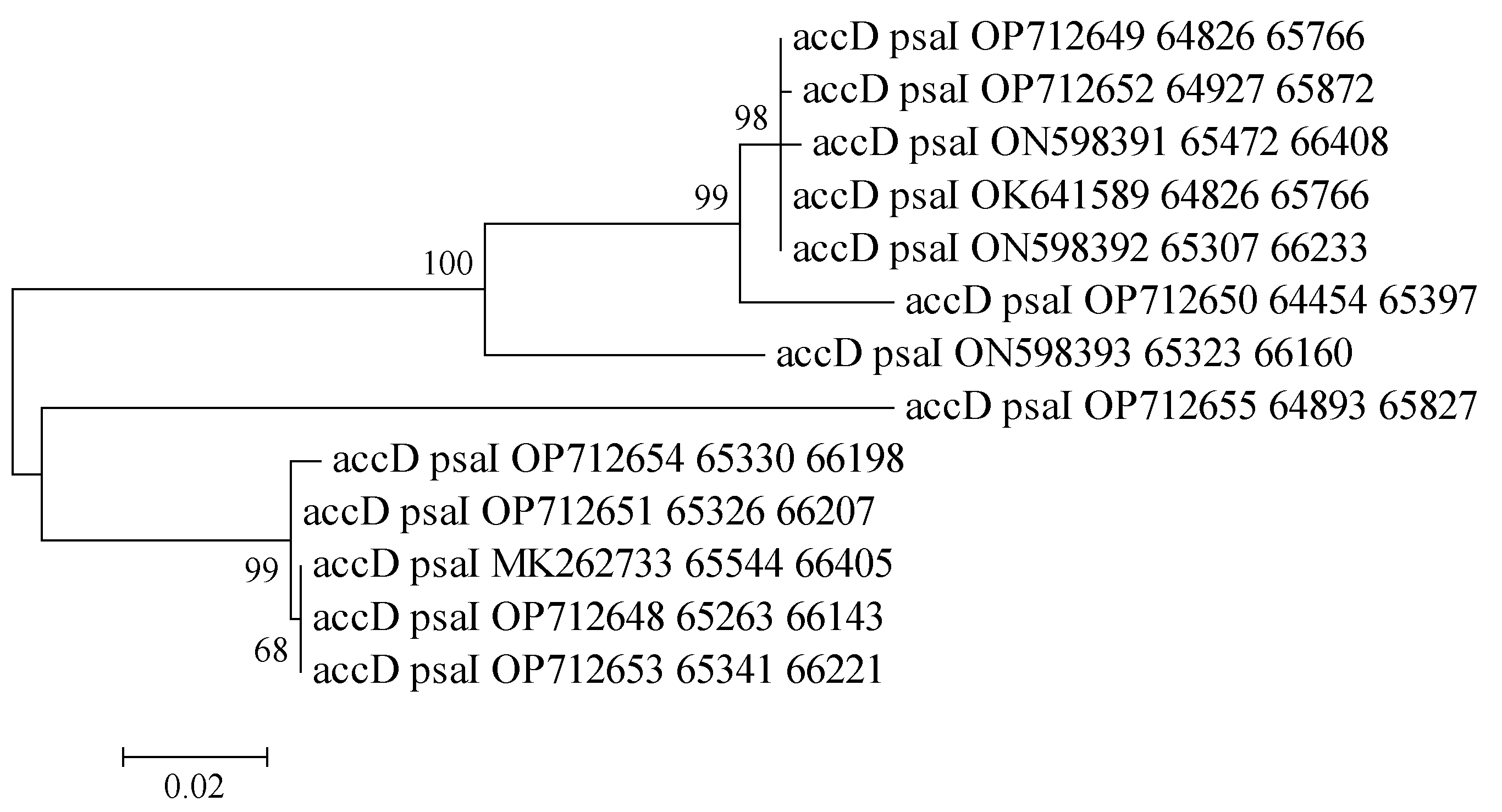


**o**





**p**

**Fig. S1** Continued.


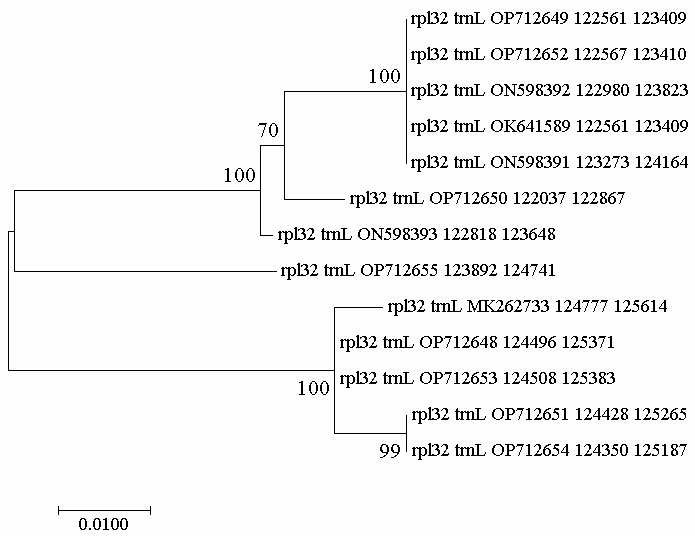


**q**





**r**


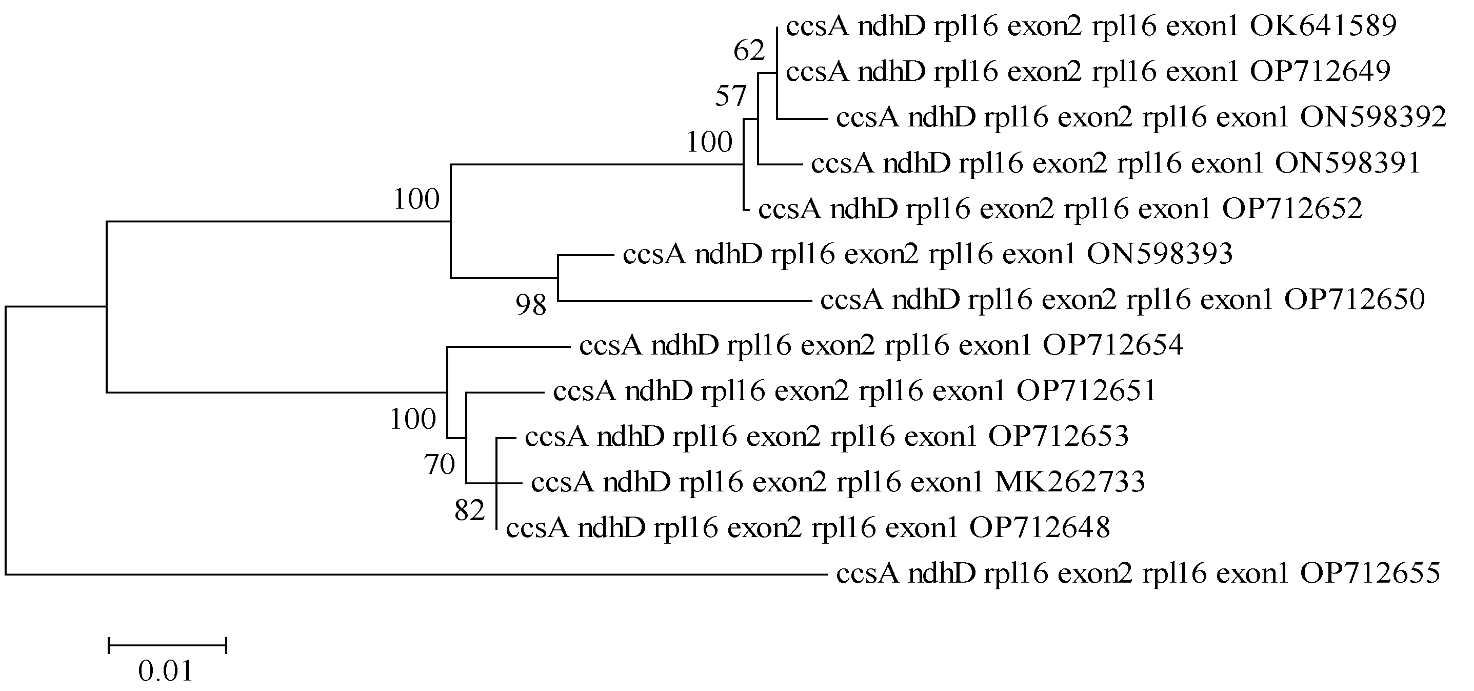


**s**

**Fig. S1** Continued.


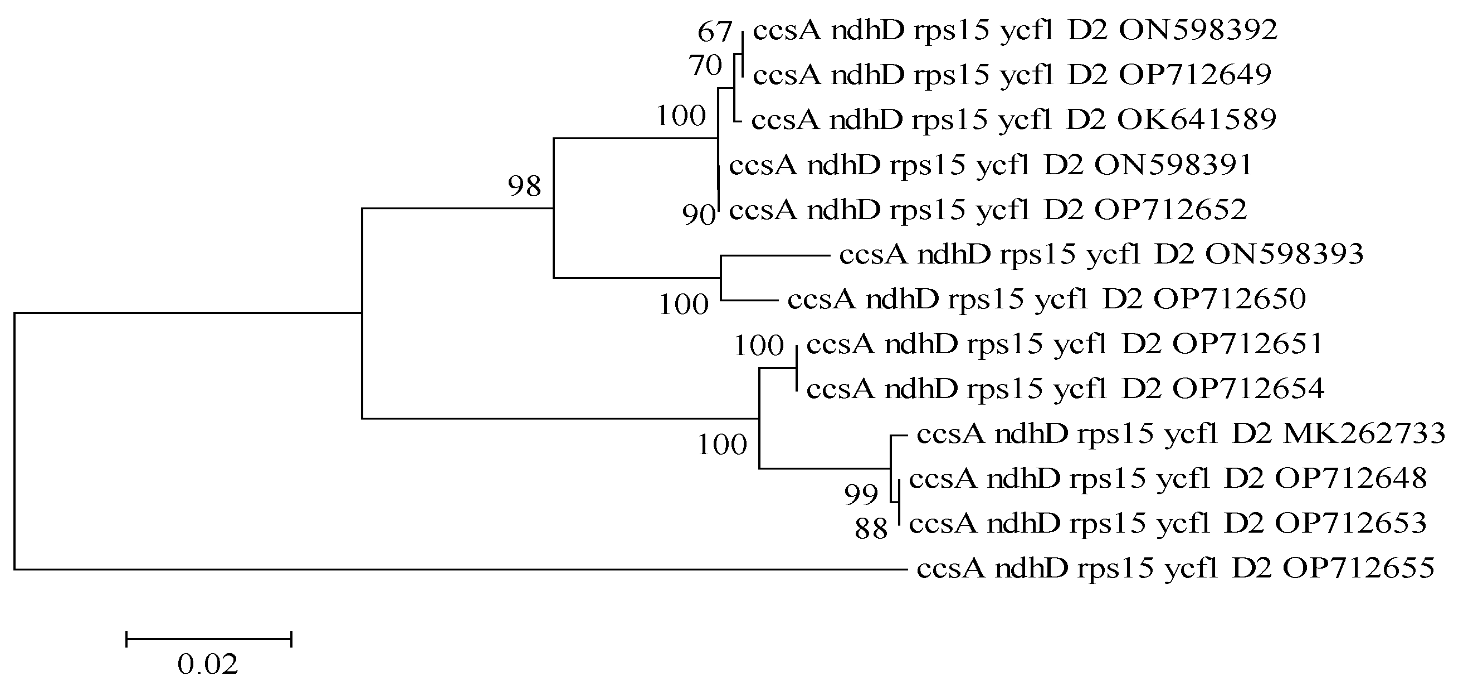

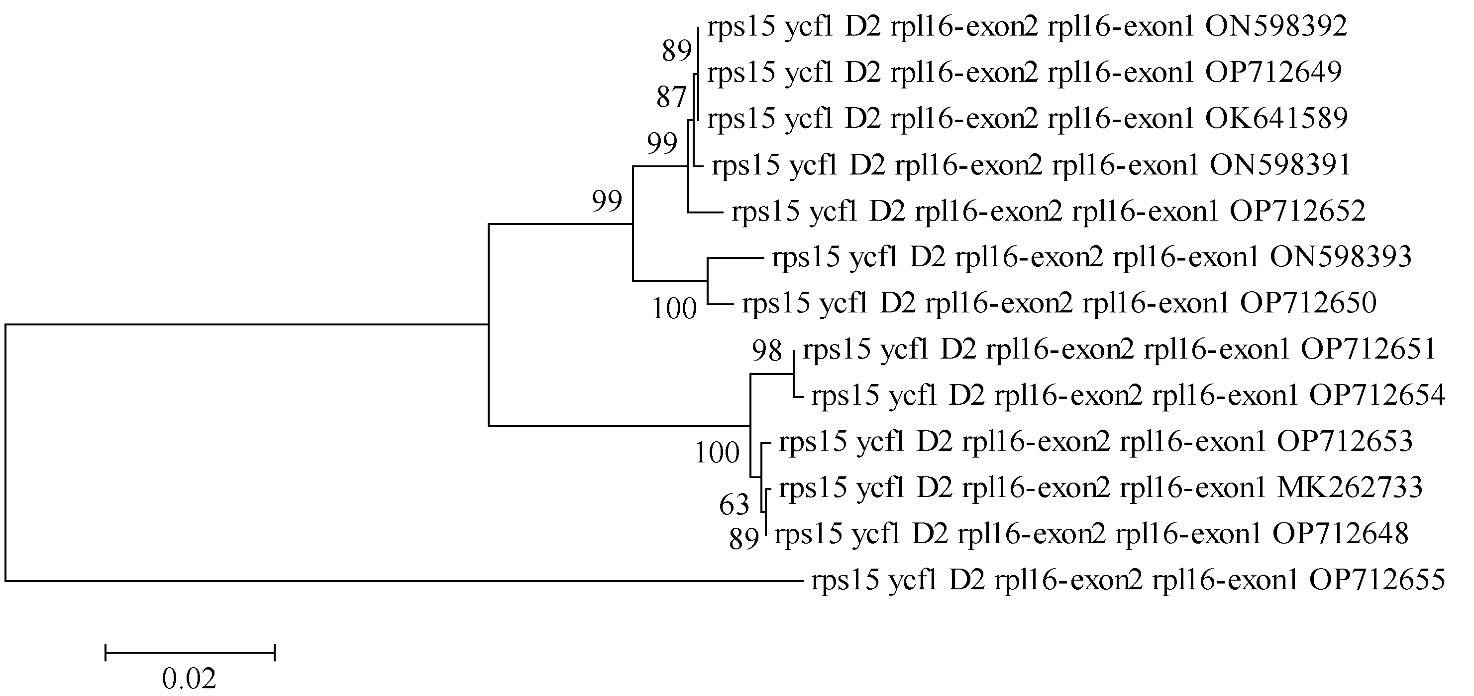


**u**

**t**


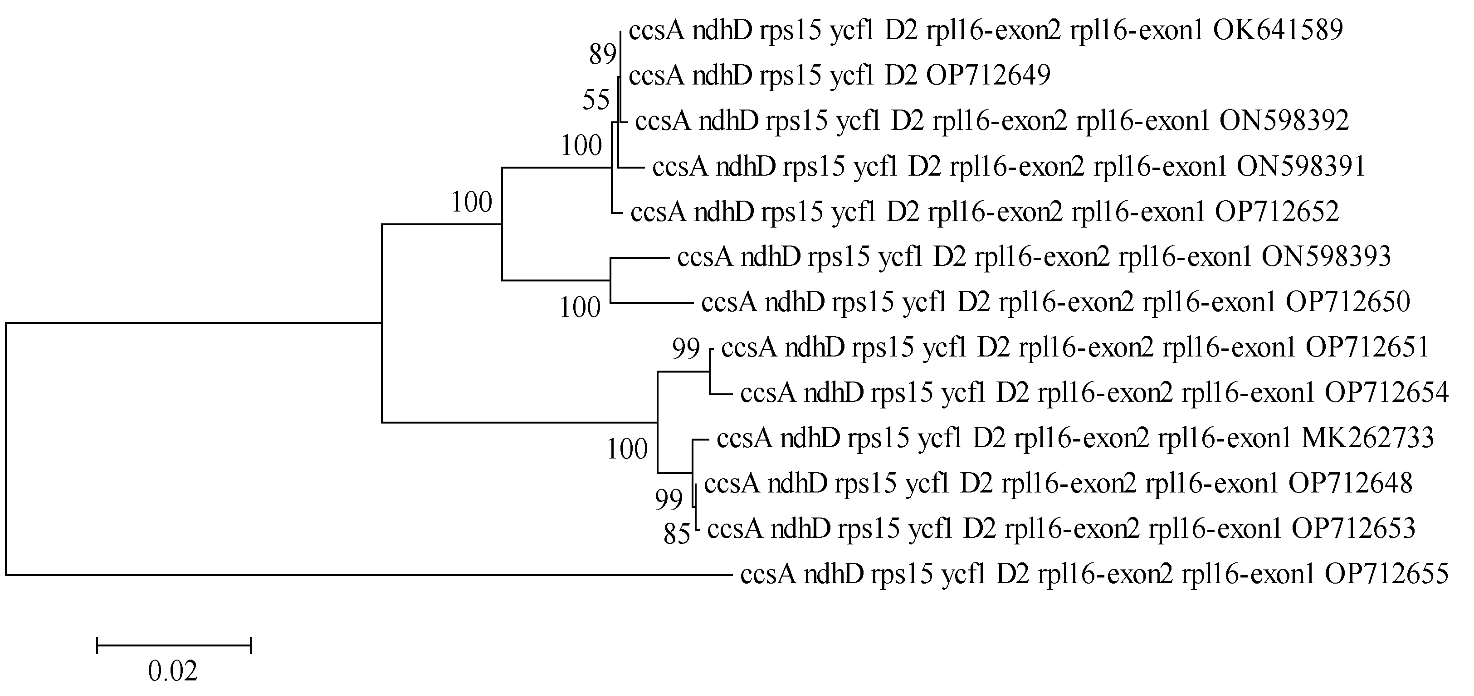


**v**

**Fig. S1** Continued.


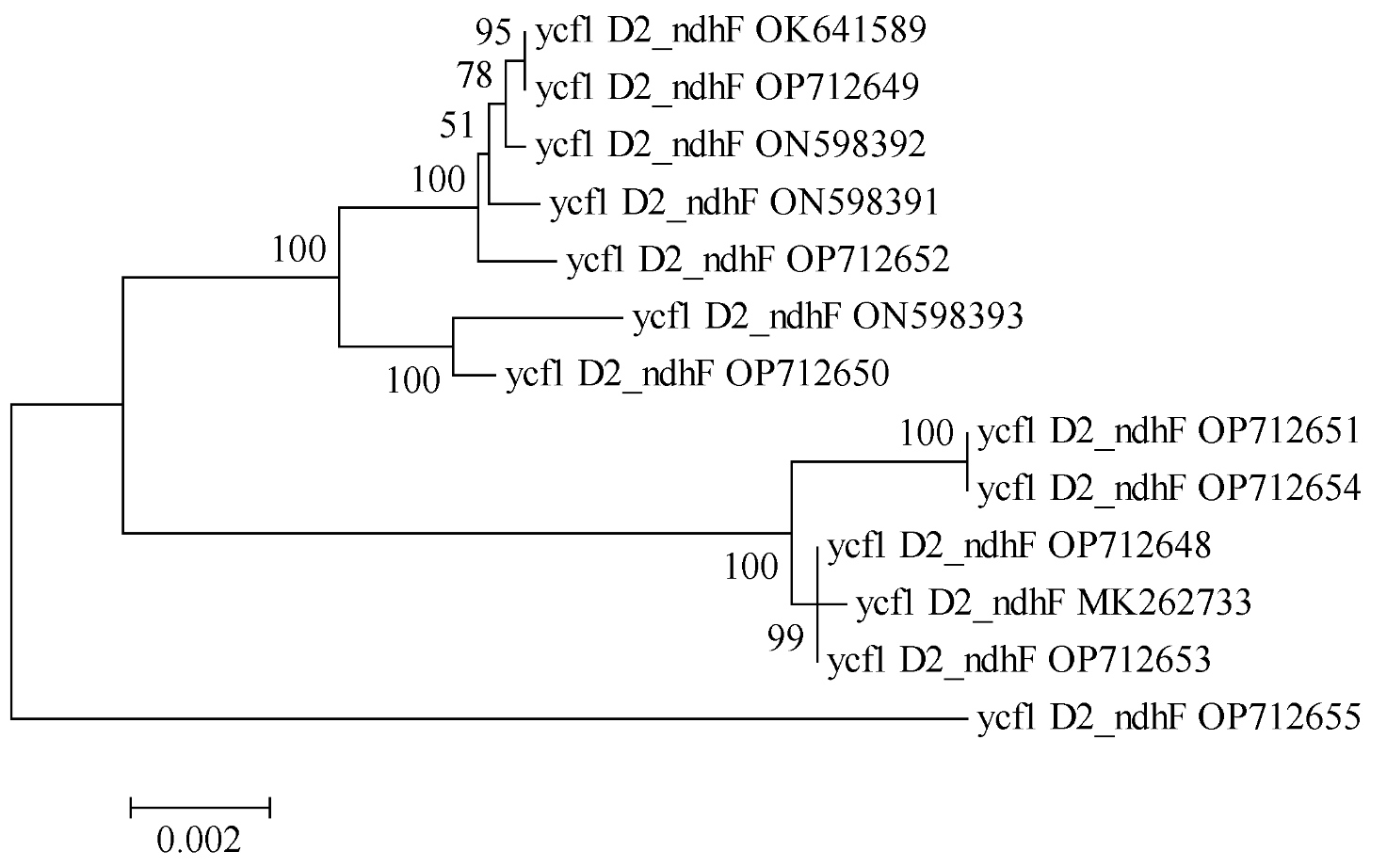


**w**

**Fig. S1** Continued.
