# Supplementary material for: Thirteen complete chloroplast genomes of the costaceae family: insights into genome structure, selective pressure and phylogenetic relationships
Source: BMC Genomics. 2024 Jan 17;25:68. doi: 10.1186/s12864-024-09996-4 (PMC10792896; doi:10.1186/s12864-024-09996-4)
Supplement: Supplementary file 12 — Supplementary Material 12: Fig. S2. Comparison of morphologies among eight species of family Costaceae. (A) terminally flowering of Costus barbatus, (B) terminally flowering of Costus speciosus Guangdong, (C) leaf morphology of Costus tonkinensis Yunnan, (D) basally flowering of Costus dubius, (E) leaf morphology of Costus speciosus var. marginatus, (F) terminally flowering of Costus woodsonii, (G) basally flowering of Costus beckii, (H) terminally flowering of C. beckii, and (I) flowering of Monocostus uniflorus [file 12864_2024_9996_MOESM12_ESM.docx]

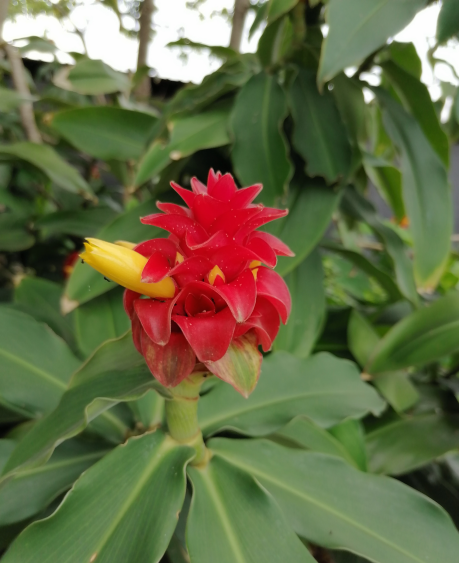

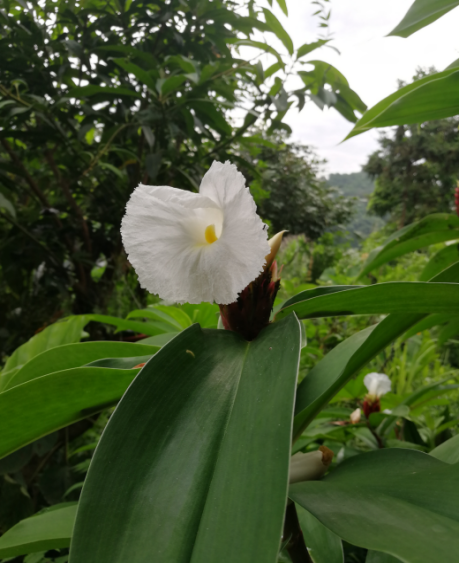

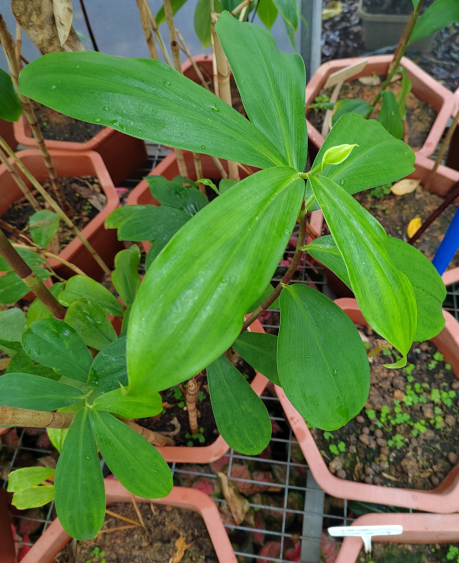


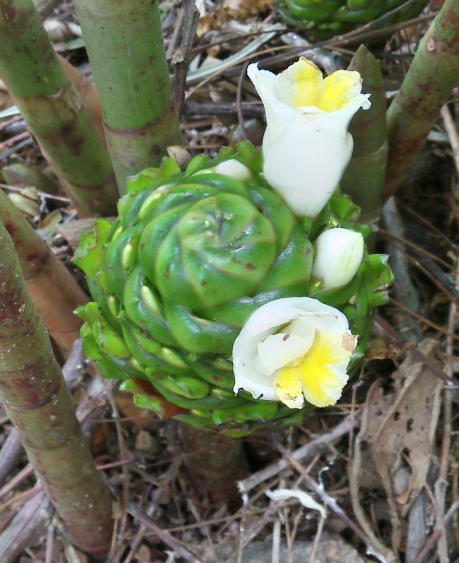

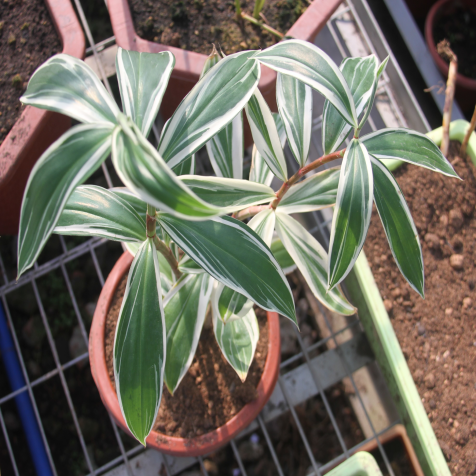

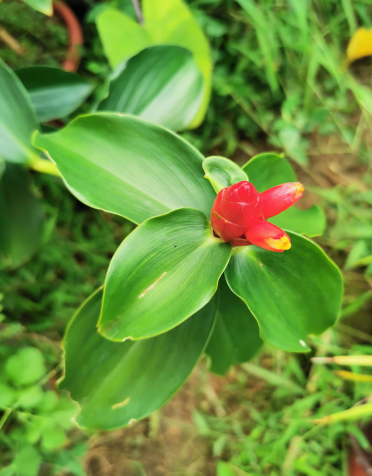


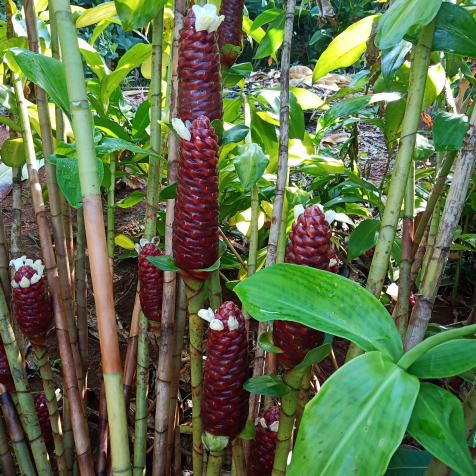

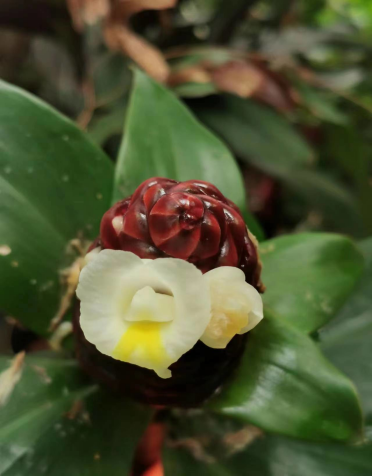

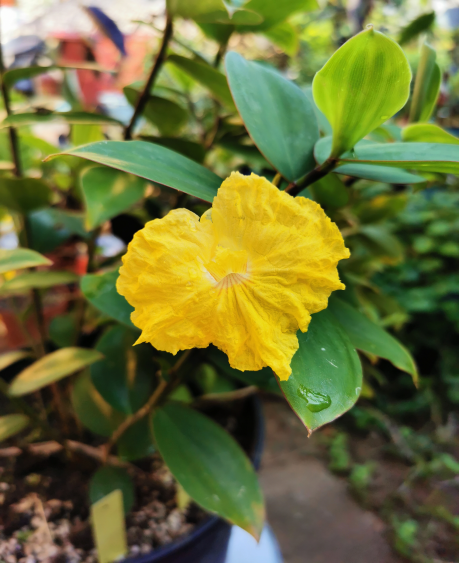


C

A

B

E

F

D

G

H

I

**Fig. S2.** Comparison of morphologies among eight species of family Costaceae. (**A**) terminally flowering of *Costus barbatus*, (**B**) terminally flowering of *Costus speciosus* Guangdong, (**C**) leaf morphology of *Costus tonkinensis* Yunnan, (**D**) basally flowering of *Costus dubius*, (**E**) leaf morphology of *Costus speciosus* var. *marginatus*, (**F**) terminally flowering of *Costus woodsonii*, (**G**) basally flowering of *Costus beckii*, (**H**) terminally flowering of *C. beckii*, and (**I**) flowering of *Monocostus uniflorus*.
